# The intracellular domains of the DSL ligands Serrate and Delta provide different activities

**DOI:** 10.1186/s12964-025-02472-w

**Published:** 2025-10-31

**Authors:** Ekaterina Seib, Maya Schmid, Hideyuki Shimizu, Tobias Troost, Sunday Faith Oyelere, Biswajit Chakraborty, Martin Baron, Thomas Klein

**Affiliations:** 1 Institute of Genetics, Heinrich-Heine-University Duesseldorf, Universitaetsstr. 1,, Duesseldorf, 40225 Germany; 2 School of Biological Sciences, University of Manchester, Michael Smith Building, Oxford Road, Manchester, M13 9PT UK

**Keywords:** Notch-pathway, Serrate, Ser, Delta, Dl, DSL-ligands, Endocytosis, E3-ligases, Mindbomb1, Mib1, Ubiquitylation, Cis-inhibition

## Abstract

Endocytosis of the ligands is a central requirement for the correct activation of the Notch-signalling pathway. It is initiated by E3-ligase mediated ubiquitylation (ubi) of the intracellular domain (ICD) of the ligands on lysine (K). In *Drosophila*, two ligands are present, termed Serrate (Ser) and Delta (Dl). They are ubiquitylated by the E3-ligases Mindbomb1 (Mib1) and Neuralized (Neur). Here, we show that the ICDs of Dl and Ser have different activities. We characterised the properties of the Ser-ICD and focused on the meaning of its Ks for ubi and signalling activity. For this purpose, we generated a variant in which all Ks of its ICD are replaced by the structurally similar arginine (R), termed SerK2R. Its analysis revealed that, in contrast to Dl, the Ks are essential for the endocytosis and degradation of Ser by Mib1. Moreover, whereas Dl possesses an ubi- and Mib1-independent signalling activity, Ser-signalling completely depends on Mib1-dependent ubi. We found that a minimum of five conserved Ks in the ICD are required for the Mib1-mediated activation of Ser and that the importance of the individual Ks differs. These core Ks appear to preferentially channel Ser into a rare signalling-relevant endocytosis pathway. Remarkably, the addition of a sixth K largely restores also bulk endocytosis, which is irrelevant for signalling. Thus, at least 6 of the 10 Ks of the ICD are required for the complete activity/behaviour of Ser.

**Supplementary Information:**

The online version contains supplementary material available at 10.1186/s12964-025-02472-w.

## Introduction

Endocytosis of the ligands plays a pivotal role during the initiation of Notch-signalling [1]. If endocytosed, the trans-interacting ligand appears to exert a mechanical (pulling) force on Notch that changes its conformation and exposes the S2-cleavage site to the cleavage by the Metalloprotease Kuzbanian (Kuz)/ADAM10. The emerging truncated Notch fragment, termed Notch EXtracellular Truncation (NEXT), is still anchored in the plasma membrane, but immediately undergoes S3-cleavage in the intramembrane region, executed by γ-secretase [2]. The S3-cleavage releases the intracellular domain of the Notch receptor into the cytosol from which it migrates to the nucleus. There, it complexes with Su(H) and transcriptional co-factors, such as Mastermind, to activate the expression of the Notch target genes. An important target gene during *Drosophila* wing development is *wingless* (*wg*) whose expression is induced along the dorso-ventral compartment boundary (D/V-boundary) of the wing imaginal disc from the third larval instar stage onwards [3]. In *Drosophila*, the core components of the pathway are the Notch-receptor, ligands of the DSL-transmembrane protein family and a transcription factor of the CSL-family, termed Suppressor of Hairless (Su (H)). In the genome of *Drosophila*, two DSL-ligands are present, termed Delta (Dl) and Serrate (Ser). Their interaction with Notch in trans, elicits the two sequential proteolytic cleavages of the receptor. Besides the productive interaction in trans, the ligands also engage in inhibitory cis-interactions with the Notch receptor. This cis-inhibition (CI) is a cell-autonomous regulatory mechanism often used to achieve directional signalling [4].

We have recently shown that the ICD of Dl is not required for the transport of Dl to the plasma membrane (exocytosis), but solely for its endocytosis [5, 6]. Endocytosis of the ligands can be initiated by ubiquitylation (ubi) of their intracellular domain (ICD). During this process, Ubiquitin is covalently attached to lysine residues (Ks) by the action of the E3-ligases Mindbomb1 (Mib1) and Neuralized (Neur) [1, 6–11]. Both ligases are important for ligand-dependent signalling. The ubiquitylated ligands can enter two endocytosis pathways, bulk endocytosis, which comprises the major fraction of Ser and Dl, but is not relevant for signalling and the Epsin-mediated pathway, which is essential for signalling, but comprises only a very small (nearly undetectable) fraction, of the endocytosed ligands [1, 6]. Epsin is a ubiquitously expressed endocytic adapter encoded by *liquid facets* (*lqf*) in *Drosophila* [12]. It possesses two Ubiquitin Interacting Motifs (UIMs) to recognise substrates such as the ubiquitylated ligand and also C-terminally located motifs that bind to the core endocytic machinery [1]. A comprehensive structure-function analysis revealed that the UIMs are important for the function of Epsin and the interaction with its ubiquitylated cargo [12, 13]. By using a knock-in allele of *Dl*, encoding a variant that cannot be ubiquitylated because all the Ks in its ICD are replaced by arginines (Rs), termed DlK2R, we recently found that the Ks of Dl are ubiquitylated by Mib1 and required for efficient endocytosis and signalling in a Lqf/Epsin-dependent manner [5, 14]. Moreover, a clear positive correlation exists between the levels of a ligand at the plasma membrane and the strength of CI, strongly supporting the notion that the interaction at the membrane in cis is the cause of CI [5, 6, 8, 14, 15]. While Mib1 is important for the full signalling activity of Dl, we recently found evidence that the Mib1 independent bulk endocytosis of Dl might be initiated by another unidentified E3-ligase, or occurs in an ubi-independent manner [14]. We previously showed that Mib1 binds to two epitopes in the ICDs of the human Ser-ortholog JAGGED1 (JAG1) and also Dl. These epitopes are termed N-box and C-box (NB and CB, respectively) and also Dli2 and Dli3 respectively [5, 9, 16].

In the case of Ser, the contribution of the two endosomal routes to signalling is revealed by the difference in the reduction of the endocytosis rate upon inactivation of *mib1* and *lqf* function. While in both mutant backgrounds the signalling activity of Ser is abolished, Ser-endocytosis is virtually absent upon loss of *mib1* function, but hardly affected by the loss of *lqf *function [6, 7, 17]. Consequently, Ser strongly accumulates in the plasma membrane of *mib1* mutant cells, but not in *lqf* mutant cells. These results indicate that, in contrast to Dl, endocytosis of Ser is generally initiated by Mib1-mediated ubi and that the decision to enter which of the two endocytic pathways is made after ubi.

 In the wing imaginal disc of *Drosophila*, Mib1 is ubiquitously expressed, whereas Neur is only expressed only in a few single late arising neural precursor cells [7, 18, 19]. Consequently, almost all ligand-dependent Notch signalling is mediated by Mib1. In general, it is found that Mib1 is largely involved in the non-neural processes, such as wing and leg development, whereas Neur is restricted to neural processes, e. g. the selection of neural precursor cells of the peripheral and the neuroblasts of the central nervous system [7].

Several studies have demonstrated the importance of ubi of the Ks in the ICD for the full activity of the ligands in Mib1-dependent processes [20]. For the mammalian ortholog of Dl, Delta-like1 (Dll1), it has been shown that K613 of its ICD appears to be crucial for its signalling activity [21]. A recent study identified 6 of the 12 Ks in the Dl-ICD as important for its signalling activity [5]. These findings indicate that ubi of several Ks is required for the full activity of Dl. However, recent investigations also showed that Dl possesses considerable activity which is independent of ubi [8, 14, 22]. Remarkably, a knock-in allele encoding DlK2R is sufficient to support the complete development, even if it is the only copy of Dl in the genome [14]. Moreover, endocytosis of DlK2R is only slightly reduced, but not abolished [8, 14, 22].

A further important result of the analysis was that the Ks in the ICD of Dl are required to suppress/adjust the level of CI by regulating the levels of the ligand in the plasma membrane: the less endocytosis occurs, the higher the levels at the surface and CI. In agreement with this observation, a recent study analysing the binding of Mib1 to Dl showed that Dl-variants that are deficient for Mib1-binding cannot be activated by Mib1 and behave very similar to DlK2R, highlighting the importance of ubi by Mib1 in CI [5]. Moreover, CI is dramatically increased in *mib1 *mutant cells [8]. In the case of Ser, replacement of two highly conserved Ks of its ICD to alanine (A) at positions 1269 and K1287, resulted in a complete inactivation of the ligand, suggesting the requirement of these two Ks for function [15]. In summary these results indicate that ubi of Ks by Mib1 suppresses and thereby regulates the level of CI.

Here, we compared the activity of the ICD of Ser with that of Dl in Mib1-dependent signalling. We found that the ICD of Ser differs in its activity from that of Dl and imposes a different endocytic behaviour onto ligands. While it is able to mediate signalling, it is more cis-inhibitory than the ICD of Dl. We subsequently focused on the meaning of the Ks in the ICD of Ser for its Mib1-dependent activation. The analysis revealed that, in contrast to Dl, the Ks are not only required, but essential for activation. Similar to Dl, they are required for endocytosis, degradation and adjustment of CI. We found that 6 of the 12 Ks in the ICD are required for the normal behaviour of Ser. However, the importance of these individual Ks differs. The 5 most conserved Ks preferentially restore its signalling activity, but not its endocytic behaviour. This suggests that they are preferentially required for the rare endocytic pathway relevant for signalling, but are not sufficient to restore bulk endocytosis. The addition of a sixth (less conserved) K appears to restore also bulk endocytosis, suggesting that it collaborates with the other 5 Ks in this pathway. Our study reveals and characterises significant differences in the behaviour and mode of activation of the founders of the two subclasses of the DSL-ligand family, Dl and Ser. It further confirms that the rate of endocytosis positively correlates with the signalling activity and negatively with the ability of a ligand to cis-inhibit. Altogether, the analysis reveals the rules of ligand-dependent Notch-signalling.

## Results

### Different activities of the ICDs of Dl and Ser

The ICDs of Dl and Ser share no significant sequence similarity [9]. This holds true also for the human orthologs of Dl and Ser (see sequence comparison in Fig. S1A). Despite this disparity, Ser and Dl can activate the Notch-pathway during development of *Drosophila* in a Mib1-dependent manner [23–25]. However, their ability to do so differs. For example, during wing development Dl can activate the pathway in the dorsal and ventral compartments of the wing primordium, whereas the ability of Ser to do so is restricted to the ventral compartment [26–31]. This difference has been attributed to differences in their extracellular domains (ECDs): Ser is unable to bind to Notch of the dorsal compartment because its ECD is modified by the glycosyltransferase Fringe (Fng) selectively expressed there [30, 32]. Also, the previously discovered higher cis-inhibitory ability of Ser compared to Dl have been traced back to differences in their ECDs [32]. The question whether the ICDs of the two ligands mediate different activities and contribute to the properties of the ligands has not been addressed. We used *Dl*^*attP*^ to address this question [33]. *Dl*^*attP*^ is a null allele, because an attP-landing site sequence replaces exon 6. Exon 6 encodes most of Dl, including its ICD. Homozygous *Dl*^*attP*^ flies die during embryogenesis because of the development of a neurogenic phenotype, which is characterised by a hyperplasia of nervous tissue at the expense of epidermis. This neurogenic phenotype is characteristic for loss of function of genes encoding core components of the Notch-signalling pathway [14, 33, 34]. *Dl*^*attP*^ allowed us to generate a knock-in *Dl*-allele that encodes a hybrid ligand where the ICD of Dl is replaced by that of Ser (*Dl*^*attP*^*-Dl-Ser-HA*). We compared its activity with that of a previously characterised fully functional control *Dl*^*attP*^*-Dl-HA* allele, generated in the same manner [14].

**Fig. 1:**
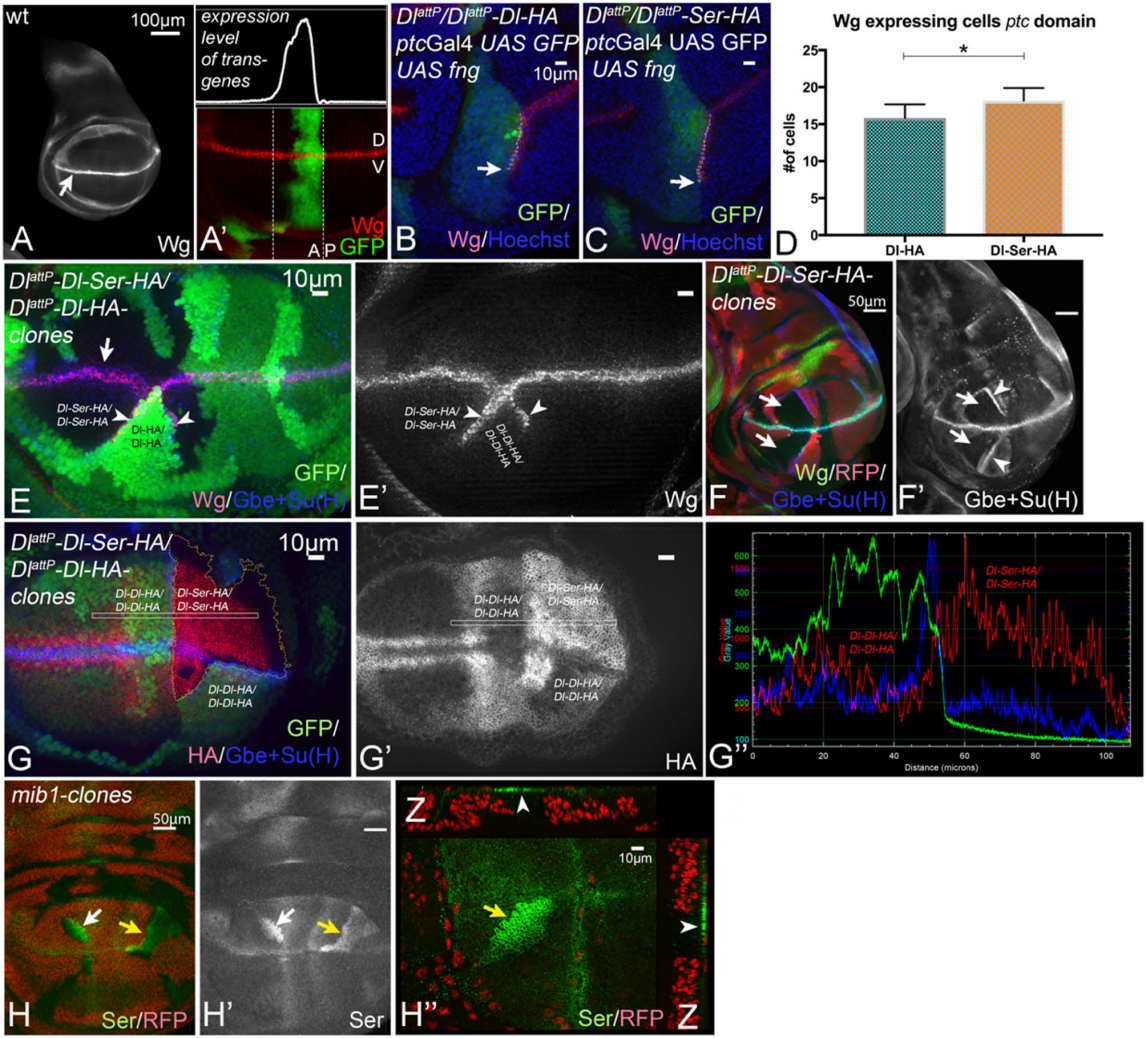
The activity of the ICD of Ser. **A**, **A’** Expression patterns of Wg (**A**) and ptcGal4 (**A’**) in a wildtype wing imaginal disc at the late third instar stage. Wg is expressed in a narrow stripe along the D/V-boundary (**A**, arrow). ptcGal4 is expressed in a band at the anterior side of the A/P boundary. In the domain, expression occurs in a gradient that increases to the A/P-boundary. Note, that the band of ptcGal4 expression runs perpendicular to that of Wg. **D**: dorsal; V: ventral. **B**-**D** Ectopic expression of Fng with ptcGal4 in Dl^attP^/Dl^attP^-Dl (**B**) and Dl^attP^/Dl^attP^-Dl-Ser (**C**) flies. It induces the ectopic expression of Wg in a stripe that straddles the A/P compartment boundary (**B**, **C**, arrow). The quantification of the length of the ectopic stripe shown in (**D**) reveals that the ectopic stripe is significantly longer in DlattP/DlattP-Dl-Ser discs (n=5). **E**-**G’’** Clonal analysis of Dl^attP^-Dl-Ser. **E**, **E’** Induction of adjacent homozygous Dl^attP^-Dl-Ser (black) and Dl^attP^-Dl (dark green) twin clones. Ectopic expression of Wg is induced in the boundary cells of Dl^attP^-Dl clones, which are located adjacent to clones homozygous for Dl^attP^-Dl-Ser (arrowheads). **F**, **F’** **A** wing disc bearing Dl^attP^-Dl-Ser homozygous clones, labelled by the absence of RFP (arrows). (**F’**) Expression of the Notch reporter Gbe+Su**(H)** is strongly suppressed in the Dl^attP^-Dl-Ser homozygous clones (arrows). The arrowheads point to the elevated expression of the Notch activity reporter in boundary cells of homozygous Dl^attP^-Dl clones. **G**, **G’** Comparison of membrane levels of the Dl-variants in adjacent homozygous Dl^attP^-Dl-Ser (black area) and Dl^attP^-Dl clones by anti-HA stainings. It reveals that the levels of Dl-Ser in the plasma membrane are higher than that of Dl. (**G’’**) Pixel density measurement in the region highlighted in (**G’**) with the rectangle. **H**, **H’’** **A** disc bearing mib1 mutant cells clones, highlighted by the arrows and labelled by the absence of RFP. Ser accumulates in the plasma membrane of the mib1 mutant cells (arrows)

*Dl* ^*attP*^ *-Dl-Ser-HA* rescued the *Dl*-mutant neurogenic phenotype of *Dl*^*attP*^ and allowed the development of the flies to the adult stage, already if it is the only Dl-variant present in the genome in one copy (*Dl*^*attP*^*-Dl-Ser-HA/Deficiency (Df)*,* (Df(3R)Dl*^*BSC850*^)). This indicates that Dl-Ser can replace Dl during *Drosophila* development. The imago displayed the expected weak dominant phenotype, such as broadening of the distal tip of the wing veins, which is typical for haplo-insufficiency of *Dl* (Fig. S1B, C, arrows). The legs, another structure very sensitive to changes in Notch activity, were wildtype in appearance in both genotypes (Fig. S1B’, C’). Nevertheless, while homozygosity of *Dl*^*attP*^*-Dl-HA* results in vital and phenotypically wildtype flies [14], nearly all homozygous *Dl*^*attP*^*-Dl-Ser-HA* flies died at the pharate adult stage. This indicates that the ICDs of Dl and Ser provide different activities and that the ICD of Ser cannot replace that of Dl. The expression pattern of *Dl*^*attP*^*-Dl-Ser-HA* was comparable to that of *Dl*^*attP*^-*Dl-HA*, indicating that it is correctly expressed (Fig. S1G, G’, H, H’). Moreover, also the expression pattern of the Notch activity reporter Gbe + Su(H) in *Dl*^*attP*^*-Dl-HA/Df *and *Dl*^*attP*^*-Dl-Ser-HA/Df* wing discs is similar, indicating that both ICDs mediate activation of Notch in the correct pattern (Fig. S1G’’, H’’).

**Fig. 2:**
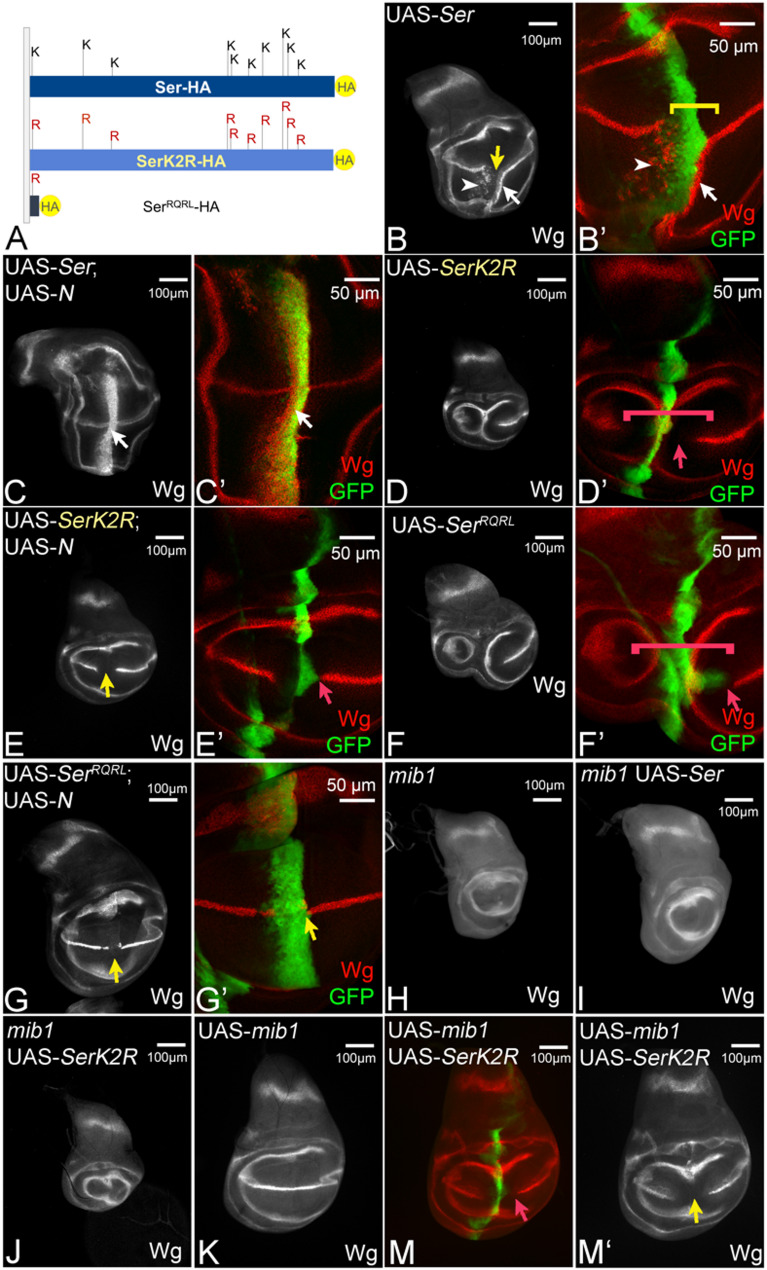
Ser requires the Ks of its ICD for Mib1-dependent signalling. **A** Cartoon of the ICDs of the Ser-variants. **B**, **B´** Expression of Ser by ptcGal4 induces ectopic expression of Wg in two stripes in the ventral compartment, (arrow and arrowhead). At high level of Ser expression, close to the A/P compartment boundary, the endogenous expression of Wg is interrupted due to CI (yellow arrow in **B** and bracket in **B’**). **C**, **C’** Co-expression of Ser with Notch results in suppression of CI, revealed by the ectopic Wg expression in the whole ptc domain (white arrow, compare with Fig. S2C). **D**, **D’**, **F**, **F’** Expression of UAS-SerK2R, or UAS-Ser^RQRL^, fails to ectopically induce the expression of Wg and interrupts its endogenous expression along the D/V-boundary at the intersection point (bracket and arrow in **D’** and **F’**). Note, that the inhibitory effect on Wg expression occurs several cell diameters aways from the ptc expression domain (highlighted by the red arrow). This non-cell-autonomous effect indicates that SerK2R and Ser^RQRL^ act in a dominant-negative manner. **E**, **E’**, **G**, **G’** Co-expression of SerK2R or Ser^RQRL^ with Notch results only in the mutual suppression of the effects caused by each construct alone, but not to ectopic expression (yellow arrow, compare with Fig. 2S1C). H A mib1 mutant wing imaginal disc. The expression of Notch along the D/V-boundary is lost due to the loss of Notch-signalling (compare with Fig. 1A, arrow). **I**, **J** Expression of Ser (**I**) and SerK2R (**J**) in a mib1 mutant discs fails to initiate ectopic Notch-signalling. **K** Over-expression of Mib1 has no effect on the expression of Wg along the D/V-boundary. **M**, **M’** Co-expression of Mib1 with SerK2R results in a phenotype that resembles that of expression of SerK2R alone. The red arrow in (**M**) points to the non-cell-autonomous interruption of the expression of Wg. It indicates that the additional over-expression of Mib1 has no effect of the activity of SerK2R. The yellow arrow in (**M’**) highlights the gap in the expression of Wg

The wing phenotype of *Dl*^*attP*^*-Dl-Ser-HA/Df* flies tended to be weaker than that of *Dl*^*attP*^*-Dl-HA* flies, suggesting that the ICD of Ser might provide slightly more signalling activity in Mib1-dependent processes (compare Fig. S1B with C, arrows). To rigorously test this notion, we monitored the signalling activities of Dl and Dl-Ser at an ectopic boundary of Fng-expressing and non-expressing cells (Fng-boundary). The ectopic expression of Fng with *ptc*Gal4 results in the ligand-dependent activation of the Notch-pathway at the posterior boundary of its ectopic expression domain in the ventral compartment of the wing disc, indicated by the induction of an ectopic stripe of Wg expression [28, 30, 35] (Fig. 1A`, B, arrow in B). The ectopic activation of the Notch pathway is sensitive to levels of Dl expression. This is revealed by a reduction of the length of the ectopic stripe in *Dl* heterozygous compared to wildtype flies [14, 36]. We monitored the expression of the endogenous target gene *wingless* (*wg*), which requires high Notch activity for its expression. Wg is expressed in a small stripe that straddles the dorso-ventral compartment boundary (D/V-boundary), which is also the expression boundary of the dorsally expressed Fng (Fig. 2A, arrow). We found that the length of the ectopic stripe of Wg induced by ectopic Fng expression is significantly longer in *Dl*^*attP*^*-Dl-Ser-HA/Df* wing discs compared to *Dl*^*attP*^*-Dl-HA/Df* discs (Fig. 1B, C, arrow, quantification in D). This finding indicates that the ICD of Ser is a more potent activator of a DSL-ligand than the Dl-ICD and explains the observed weaker haplo-insufficient vein phenotype of adult *Dl*^*attP*^*-Dl-Ser-HA*/*Df* compared to *Dl*^*attP*^*-Dl-HA*/*Df* flies (Fig. S1B, C).

### The ICD of Ser mediates higher CI compared to the ICD of Dl

To further compare the properties of the ICDs of Ser and Dl, we used clonal analysis to generate cell clones homozygous for *Dl*^*attP*^*-Dl-HA* adjacent to homozygous *Dl*^*attP*^*-Dl-Ser-HA* clones (Fig. 1E, E’). We found that Wg was correctly expressed along the D/V-boundary in the area of homozygous *Dl*^*attP*^*-Dl-Ser-HA* clones, indicating that the signalling at the D/V-boundary is not interrupted and that *Dl*^*attP*^*-Dl-Ser-HA* can provide the necessary Notch signalling activity together with Ser (Fig. 1E, arrow). Interestingly, we observed that the expression of Wg is ectopically activated in boundary cells of *Dl*^*attP*^*-Dl-HA* homozygous clones, abutting the homozygous *Dl*^*attP*^*-Dl-Ser-HA* clones (Fig. 1E, E’, arrowheads). This indicates that *Dl*^*attP*^*-Dl-Ser-HA* homozygous cells are more potent signallers, but less potent signal-receivers, compared to the adjacent *Dl*^*attP*^*-Dl-HA* homozygous cells and can elicit ectopic Notch signalling in *Dl*^*attP*^*-Dl-HA* homozygous cells. This behaviour is typical for cells expressing a strongly cis-inhibitory ligand, suggesting that Dl-Ser-HA is probably more cis-inhibitory than Dl-HA. This conclusion is strongly supported by the observation that the expression of the sensitive Notch activity sensor Gbe + Su(H) was dramatically suppressed within *Dl*^*attP*^*-Dl-Ser-HA* homozygous clones in comparison to homozygous *Dl*^*attP*^*-*Dl-HA clones and heterozygous *Dl*^*attP*^*-Dl-Ser-HA*/*Dl*^*attP*^*-Dl-HA* territories (Fig. 1F, F’, arrows). As in the case of Wg, the sensor was upregulated in homozygous *Dl*^*attP*^*-Dl-HA* boundary cells (Fig. 1F’, arrowheads). In our previous analysis of Dl, we found that the degree of CI is determined by the levels of the ligand in the plasma membrane [8, 14]. Thus, a possible reason for the observed stronger cis-inhibitory abilities of Dl-Ser-HA is that it is less efficiently endocytosed compared to Dl-HA and therefore present at the plasma membrane at higher levels [14]. To investigate this possibility, we monitored the subcellular localisation of Dl-Ser-HA and Dl-HA in adjacent homozygous clones by anti-HA staining (Fig. 1F-F’’). We found that Dl-Ser-HA accumulated to higher levels in the plasma membrane compared to Dl-HA in the clone experiments (Fig. 1G-G’’). Combined with the findings that the ICD of Ser is not required for the delivery of Ser to the plasma membrane (see below), but for its endocytosis and that the increased accumulation of Dl-Ser in the plasma membrane resembles the accumulation of Ser in *mib1* mutant cells, where the endocytosis of Ser is strongly suppressed [5, 7, 14] (see also Fig. 1H-H’’, arrows and arrowheads), these findings strongly suggest that the difference in activity of Dl-HA and Dl-Ser-HA is a result of a difference in the ability of their ICDs to mediate endocytosis, with Dl-Ser being less efficiently endocytosed. The reduced efficiency results in a higher abundance of Dl-Ser in the plasma membrane, which probably results in a higher probability to interact with Notch in cis to cis-inhibit and trans to signal. We previously showed that the Ks of the ICD of Dl (*Dl*^*attP*^*-DlK2R*) are responsible for the efficiency of endocytosis, the level of activity and CI in Mib1-mediated signalling [5, 14]. Therefore, we concentrated on the importance of the Ks in the ICD of Ser.

**Fig. 3:**
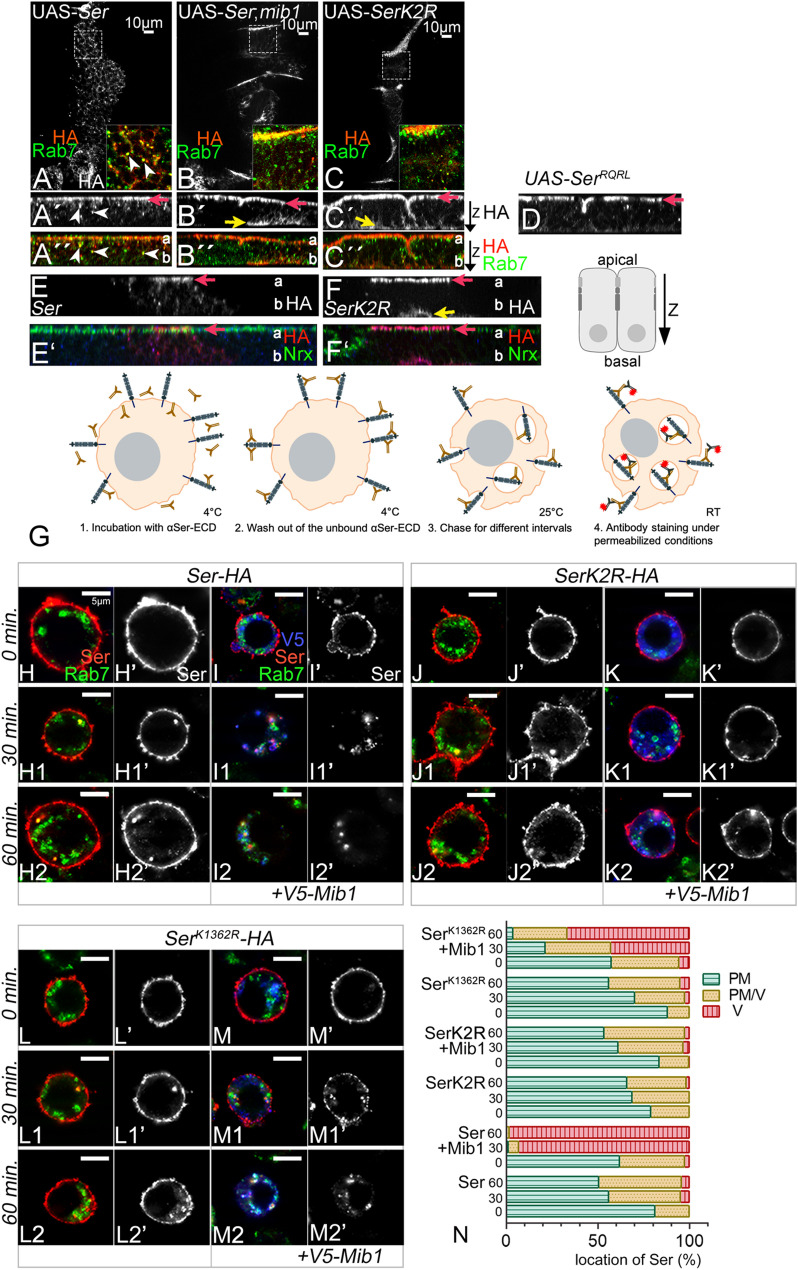
The intracellular Ks are essential for the Mib1-mediated endocytosis of Ser. **A**-**F’** Subcellular localisation of the Ser-variants revealed by anti-HA antibody staining. a: apical; b: basal. Rab7 staining was used to mark endosomes. Nrx staining in (**E**-**F’**) labels the apical side of the epithelium. **A**-**A’’**, **E**,**E’** Subcellular localisation of Ser in wildtype wing imaginal discs. Ser localises at the apical membrane and Rab7-positive endosomes (red arrow and arrowheads, respectively). The frame in A shows an enlarged view of the area marked with the rectangle. The arrowheads point to some of the Ser-HA- and Rab7-positive vesicles. **B**-**B''** In mib1 mutant cells, endocytosis of Ser is strongly reduced (see also Fig. 1**H**-**H’’**). Most of the HA signal can be observed at the apical and the basal membrane (red and yellow arrow in **B'**, respectively). Moreover, Ser is virtually absent from endosomes. Note the accumulation of Ser also in the basal membrane (yellow arrow). **C**-**C''**, **F**,**F’** SerK2R localisation in wild-type cells. Ser localises to the apical and to the basal membrane, but is hardly seen in endosomes (red and yellow arrow, respectively). **D** Expression of SerRQRL in wildtype cells results in its accumulation in the apical membrane (red arrow). This indicates that the ICD of Ser is not required for the transport of Ser to the apical membrane. **G**-**N** Antibody uptake assay to analyse the endocytosis of Ser, SerK2R and Ser^K1362R^ in S2R+ cells. **G** Design of the assay. The antibody is raised against the ECD of Ser. **H**, **I**, **J**, **K**, **L** At time point 0, all antibody labelled Ser-variants were located in the plasma membrane. (H1-H2, J1-J2, L1-L2). In absence of Mib1, Ser, SerK2R, Ser^K1362R^ were present in plasma membrane after 30 and even 60 min., indicating that they are not efficiently endocytosed in the absence of Mib1. (**I**-I2’) Co-expression of Ser with Mib1. The presence of Mib1 results in the efficient internalisation of Ser, which localises to Rab7-positive endosomes after 30 and 60 min. No Ser was observed in the plasma membrane already after 30 min. (**K**-K2’) In contrast to Ser, the presence of Mib1 does not induce the internalisation of SerK2R, indicated by the presence of SerK2R in the plasma membrane, even after a chase of 60 min. (**L**-M2’) Ser^K1362R^ can be endocytosed in the presence of Mib1. However, in contrast to Ser, a fraction is still present at the plasma after a chase of 30’, suggesting that it is less efficiently endocytosed than Ser. N Quantification of the endocytosis of the Ser-variants. For the quantification, the cells were counted in which anti Ser-ECD was detected either at the plasma membrane (PM), at the plasma membrane and in vesicles (PM/V) or in vesicles only (V). The corresponding cell number was calculated in relation to the total cell number. The analysis reveals that Ser^K1362R^ is less efficiently endocytosed than Ser, but more efficiently than SerK2R

### The Ks in the ICD of Ser are essential for its activity

The ICD of Ser contains 10 Ks, which are potential targets for Mib1-mediated ubi (Fig. 2A). Five of them are highly conserved throughout insect species (Fig. S2A). To investigate the role of the Ks, we first generated a Ser-variant, where all Ks of the ICD were replaced by the structurally similar arginine (R) (SerK2R). In contrast to Ser, ubi of SerK2R by Mib1 is strongly impaired in an ubi assay using S2 + cells, indicating that the Ks are required for ubi of Ser by Mib1 (Fig. S3A, B, lane 4 and 5). As controls in our experiments in the wing disc, we also generated a variant with the wildtype ICD and Ser^RQRL^, a variant where most of the ICD is deleted (Fig. S2A, Fig. 2A). All variants were C-terminally tagged with HA, inserted into the same genomic landing site to guarantee comparable expression levels and expressed with *ptc*Gal4. Note, that only Mib1 is present during wing development. Therefore, the observed phenotypes in the wing primordium reveal changes in Mib1-mediated activation of Ser.

**Fig. 4:**
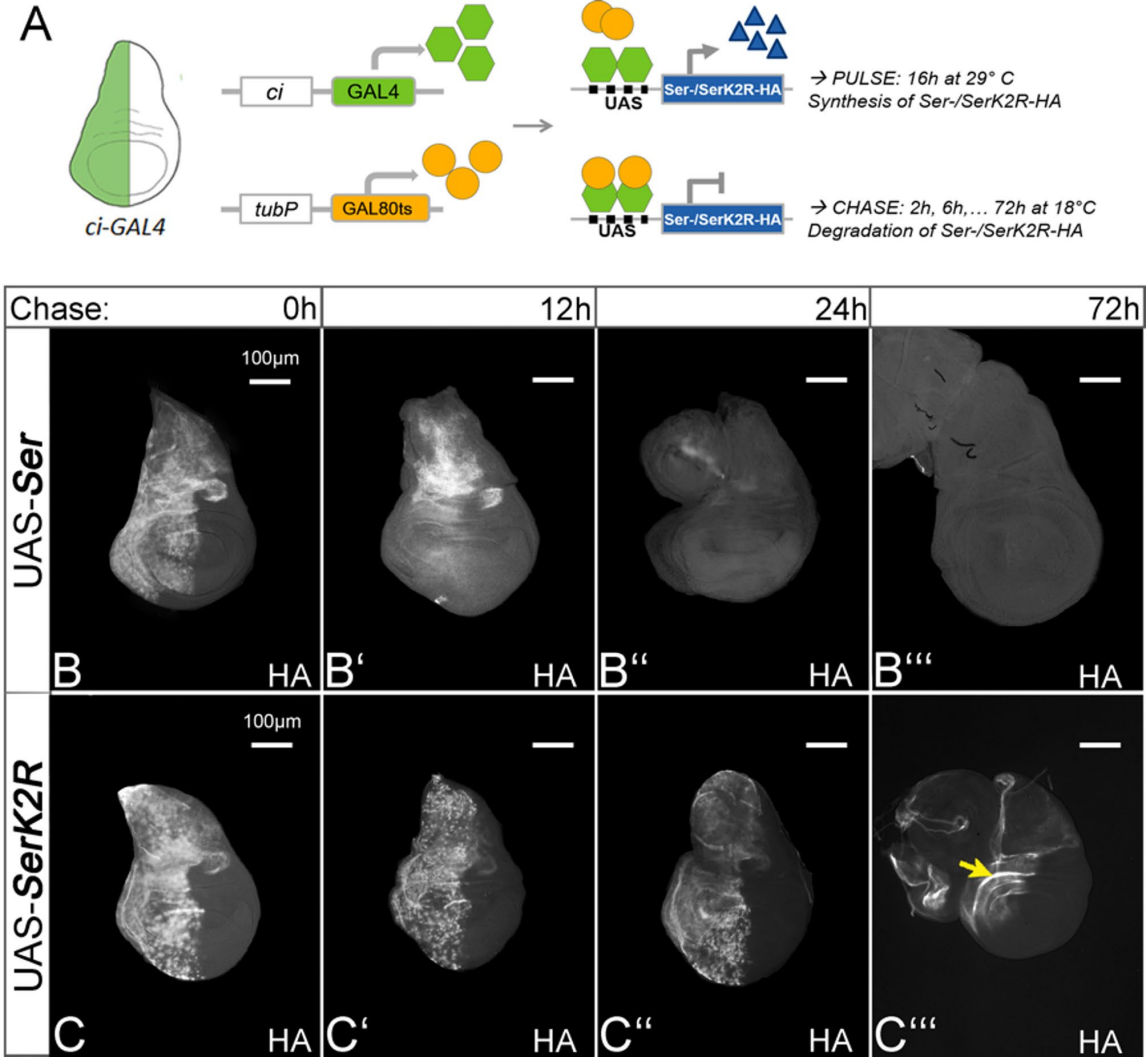
Degradation of Ser and SerK2R. **A** Design of the pulse-chase experiment. Ser-HA and SerK2R-HA were expressed under the control of ciGal4 combined with the temperature sensitive tubGAL80ts. **B**, **C** At time 0, both ligands can be detected in the whole ci-Gal4 expression domain. **B**-**B’’’** The HA signal of Ser-HA vanishes gradually within 24 hours. **C**-**C’’’** In contrast, SerK2R-HA is detectable even after 72 hours of chase (yellow arrow), indicating that it is inefficiently degraded in comparison to Ser-HA

**Fig. 5:**
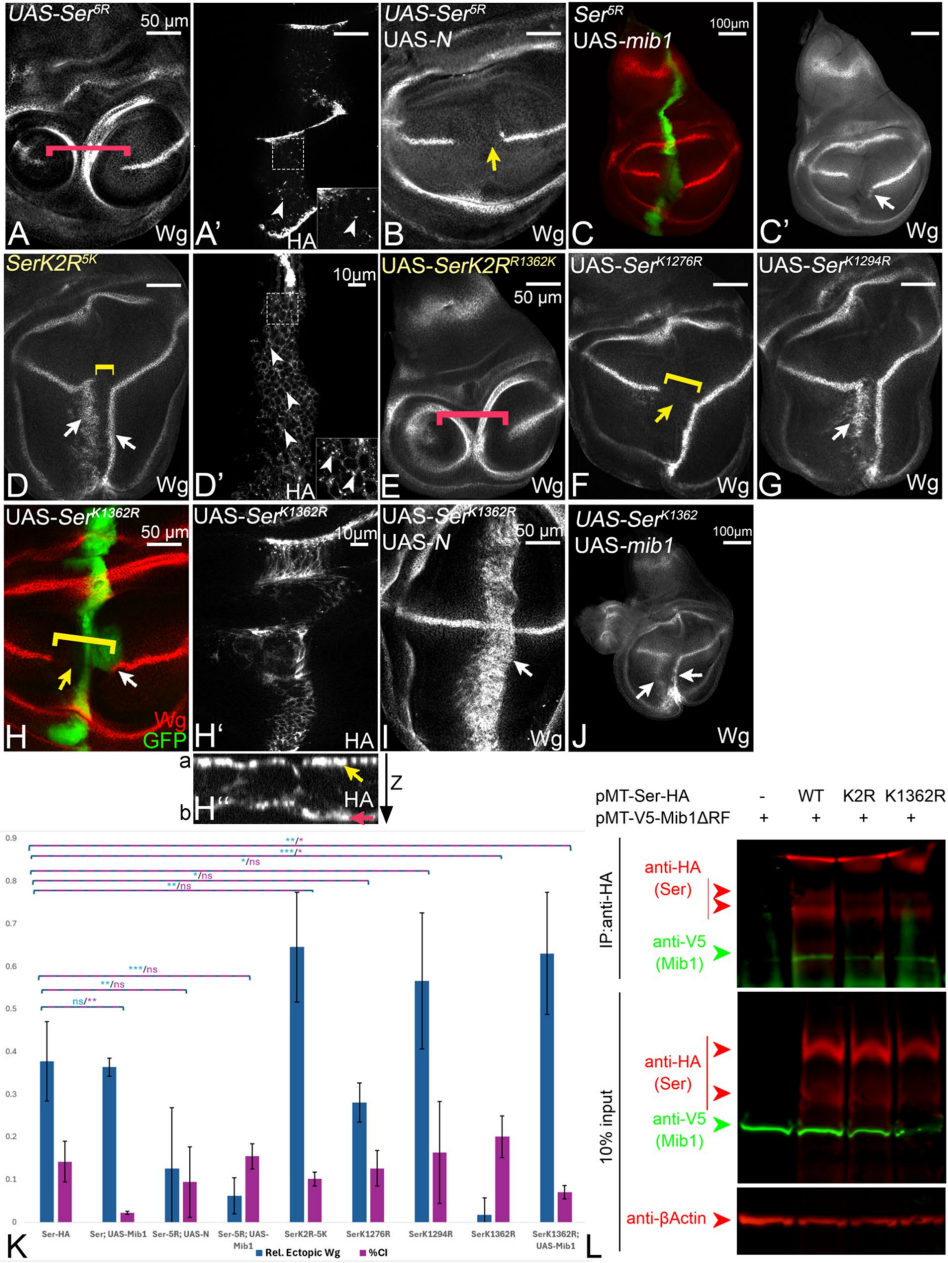
Analysis of the individual core Ks in the ICD of Ser. **A**, **A’** Expression of Ser^5R^ fails to induce ectopic expression of Wg and interrupts the endogenous expression of Wg in a manner comparable to SerK2R (red bracket, compare with Fig. 2**D**, **D’**). This indicates that it acts in a dominant-negative manner. (**A’**) Nevertheless, it is still located in intracellular punctae, suggesting that it is endocytosed. **B** Like in the case of SerK2R, the co-expression of Ser^5R^ with Notch results in the mutual suppression of the individual phenotype (compare with Fig. 2**E**, **E’**). **C**, **C’** Co-expression of Ser^5R^ with Mib1 resulted in a weak activation of signalling, indicated by the weak ectopic activation of Wg close to the D/V boundary (arrow, quantification in **K**). **D**, **D’** Expression of SerK2R^5K^ induces an ectopic activation of Wg in a manner very similar to Ser. Note, that the anterior stripe is longer than in the case of Ser, indicating a slight stronger signalling ability (quantification in **K**). SerK2R-5K is also found in intracellular punctae (arrow in **D’**). **E** The re-introduction of K1362 into the ICD of SerK2R does not impact on its activity. SerK2R^R1362K^ still displays the dominant-negative phenotype, similar to SerK2R (**B**, red bracket, compare with Fig. 2**E**, **E’**). **F** The replacement of K1276 by R in Ser produces an active ligand with a slightly higher cis-inhibitory effect, indicated by the absence of the anterior stripe of ectopic expression of Wg (yellow arrow and bracket, compare with Fig. 2**C**, **C’**). **G** Replacement of K1294 by R in Ser leads to a slightly more active ligand. The white arrow points to the anterior stripe of the ectopic Wg expression, which is slightly more prominent than that of Ser (quantification in **K**, compare with Fig. 2**B**, **B’**). **H**-**H’’** The signalling activity and endocytosis of Ser^K1362R^ is strongly impaired. Yellow arrow in **G** points to the enlarged gap of endogenous Wg expression (yellow bracket). Note, that a slight ectopic activation of Wg expression close to the D/V-boundary is detectable in a fraction of discs (white arrow). This indicates that the signalling abilities are dramatically reduced, but not abolished and that Ser^K1362R^ is not acting in a dominant-negative manner, but possesses increased cis-inhibitory abilities (quantification in **K**). (**H´´**) Similar to SerK2R, Ser^K1362R^ is located mostly at the apical (yellow arrow) and the basal membrane (red arrow). a: apical; b: basal. **I** Co-expression of Ser^K1362R^ with Notch results in suppression of Ser^K1362R^ mediated CI and in a strong ectopic activation of ectopic Wg expression, comparable to that of co-expression of Notch with Ser (white arrow, compare with Fig. 2**D**). **J** Likewise, the Co-expression with Mib1 strongly enhance the signalling abilities of Ser^K1362R^ (arrows). **K** Quantification of the signalling and cis-inhibitory abilities of the Ser-variants with the exception of the dominant-negative acting variants (n= 5 discs/genotype). **L** Co-immunoprecipitation experiments (Co-IP) to analyse the binding of Ser, SerK2R, Ser^K1362R^ to V5-Mib1ΔR, a variant without the Ring Finger domain required for ubi. The results of the Co-IP indicates that all three Ser variants are able to interact with Mib1, as they are co-precipitate Mib1

As previously reported, expression of Ser-HA induces the ectopic expression of Wg in the ventral compartment of the wing primordium in two stripes [23, 26] (Fig. 2B, B’). The more diffuse and shorter anterior stripe is located in the region of lower expression of the *ptc*Gal4 expression gradient (Fig. 2B, B’, arrowhead). The posterior, more defined, but thinner stripe is located in adjacent non-expressing posterior boundary cells (Fig. 2B, B’, white arrow). This is confirmed by the co-expression of Ser with a N-RNAi construct: In this case, the anterior stripe of ectopic Wg expression is abolished, but the ectopic posterior stripe is unaffected (Fig. S2D). In the region of high expression of Ser, close to the A/P-boundary, Notch-activation is cell-autonomously repressed due to CI (Fig. 2B, B’ yellow arrow). In the dorsal compartment, modification of Notch by the dorsally expressed Fringe prevents Ser-signalling [30]. Previous work has shown that CI of the *Drosophila* ligands can be suppressed by their co-expression with Notch [37]. As a result of this co-expression, Notch signalling is ectopically activated throughout the *ptc*Gal4 domain (Fig. 2C, C’, arrow). Ser fails to activate ectopic Wg expression in *mib1* mutant discs, confirming previous results that the activation of Notch depends on the activity of Mib1 (Fig. 2H, I). In contrast to Ser, neither SerK2R, nor Ser^RQRL^ could activate Notch ectopically in wildtype and *mib1* mutant discs (Fig. 2D, D’ and F, F’). The expression of these variants interrupted the endogenous expression of Wg in a non-cell-autonomous manner (Fig. 2D’, F’ red arrow and bracket). Moreover, co-expression of the two Ser-variants with Notch resulted in the mutual cancellation of the effects caused by expression of each construct alone, but not in ectopic expression of target genes, as observed in the case of Ser (Fig. 2E, E’, G, G’, yellow arrow, compare with Fig. S2C). The phenotypes indicate that Ser^RQRL^ and SerK2R have lost their signalling activity and act in a dominant-negative manner. Thus, SerK2R behaves similar to a Ser-variant without an ICD, indicating that the ICD of Ser becomes non-functional in Mib1-dependent processes in the absence of its Ks. This notion is further supported by gain of function experiments that showed that the over-expression of Mib1 did not change the dominant-negative behaviour SerK2R (Fig. 2K-M’, red and yellow arrow, compare with E, E’), These results highlight the requirement of the Ks for Mib1-dependent activation of Ser and strongly suggest that, for the activation, Mib1 ubiquitylates one or more Ks in the ICD of Ser. Note, that Ser behaves different from Dl, which is weakly active, even in the complete absence of Ks in its ICD [8, 14].

### The Ks of the ICD are required for Mib1-dependent endocytosis and degradation of Ser

**Fig. 6:**
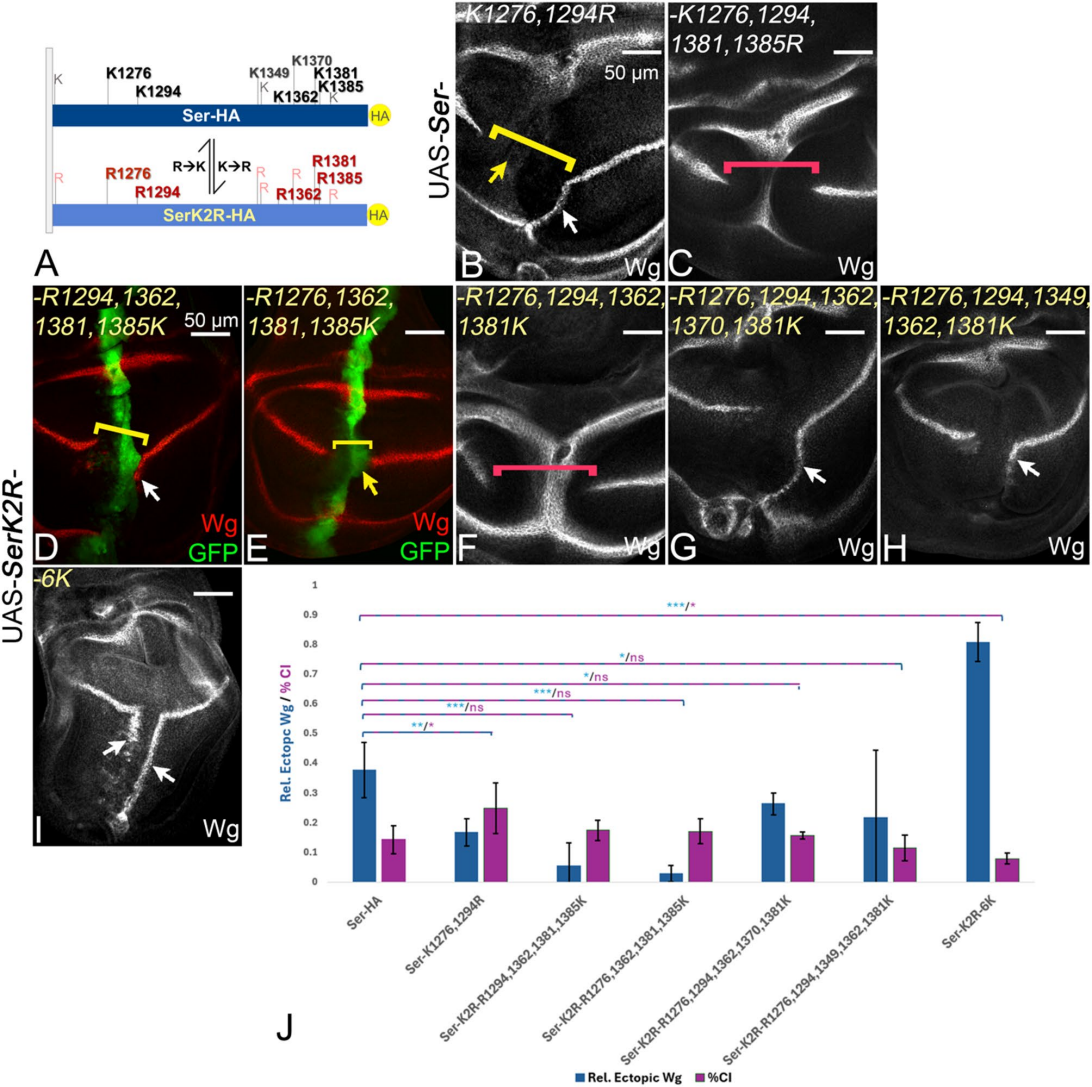
Analysis of Ser- and SerK2R-variants with multiple K to R and R to K substitutions. **A** Diagram of the ICDs of Ser and SerK2R for orientation. **B** Simultaneous replacement of K1276 and K1294 by R led to a less active ligand (white arrow) with enhanced cis-inhibitory properties (yellow arrow, bracket). **C** Exchange of four out of five core K also led in each combination to an inactive ligand (see also Fig. S4L, M). **D**-**F** Reintroduction of four out of five core Ks resulted in ligands with different activities. SerK2R^R1294,1362,1381,1385K^ was able to ectopically activate Wg only very weakly, close to the D/V boundary (**D**, white arrow). SerK2R^R1276,1362,1381,1385K^ was no longer dominant-negative, but only cis-inhibitory (E, yellow arrow) and could not induce ectopic Wg expression. SerK2R^R1276,1294,1362,1381K^ remained not functional, indicated by the lack of ectopic activation of Wg and strong CI (F, red bracket). **G** The reintroduction of alternative less conserved K1370 together with Ks 1276,1294,1362,1381 fully restores the activity of the ligand (white arrow, compare with F). **H** The re-introduction of K1349, which is similarly conserved than K1370, together with 1276,1294,1362,1381 resulted in a less active Ser variant, which only weakly activated Wg expression. **I** Expression of SerK2R^6K^, where K1370 is re-introduced with the five core Ks, results in a strong activation of ectopic Wg expression, comparable to SerK2R^5K^ (compare with Fig. 5A). **J** Quantification of the signalling and cis-inhibitory activities of the variants with the exception of the dominant-negative acting variants (n= 5 discs/genotype)

Previous work indicates that *mib1* function is essential for the endocytosis of Ser [6, 7] (Fig. 1H-H’’). In wildtype disc cells, Ser is located in the apical plasma membrane, apical to the septate junction marker Neurexin (Nrx) and on maturing endosomes [15]. This work also showed that endosomal localisation of Ser is dramatically reduced or abolished in *mib1*-mutant cells. Instead, Ser strongly accumulates in the apical plasma membrane of the disc cells, confirming the previously reported importance of Mib1 for endocytosis of Ser (Fig. 1H-H’’) [6, 7]. In agreement with these observations, we here observed that also ectopically expressed Ser localized to the apical membrane and to Rab7-positive endosomes (Fig. 3A-A’’, D, D’, red arrow and arrowheads, respectively). Likewise, in *mib1* mutant cells, ectopically expressed Ser accumulated at the plasma membrane, but was mostly absent from Rab7-positive endosomes (Fig. 3B-B’’, red arrow). We observed additional accumulation of Ser also in the basal membrane of these cells (Fig. 6B’, yellow arrow). The meaning of this observation is not clear at the moment. It might reflect a complex transport of nascent Ser to its destination at the apical membrane, as previously found for the septate junction protein Megatrachea [38]. We found that in wildtype discs SerK2R, behaves very similar to Ser in *mib1* mutant discs: it accumulated in the apical membrane of the disc cells, near the septate Junction constituent Nrx, and was hardly detectable on endosomes (Fig. 3B-B’’’, F, F’, red arrow). Note, that Ser^RQRL,^ which lacks the ICD, also localised in the apical membrane, indicating that, just like in the case of Dl, the ICD is not required for the transport of Ser to the apical membrane (Fig. 3D, red arrow). Thus, the loss of its Ks in the ICD appears to strongly reduce the efficiency of endocytosis of Ser mediated by Mib1. As a consequence of this reduction, it accumulates in the plasma membrane.

**Fig. 7:**
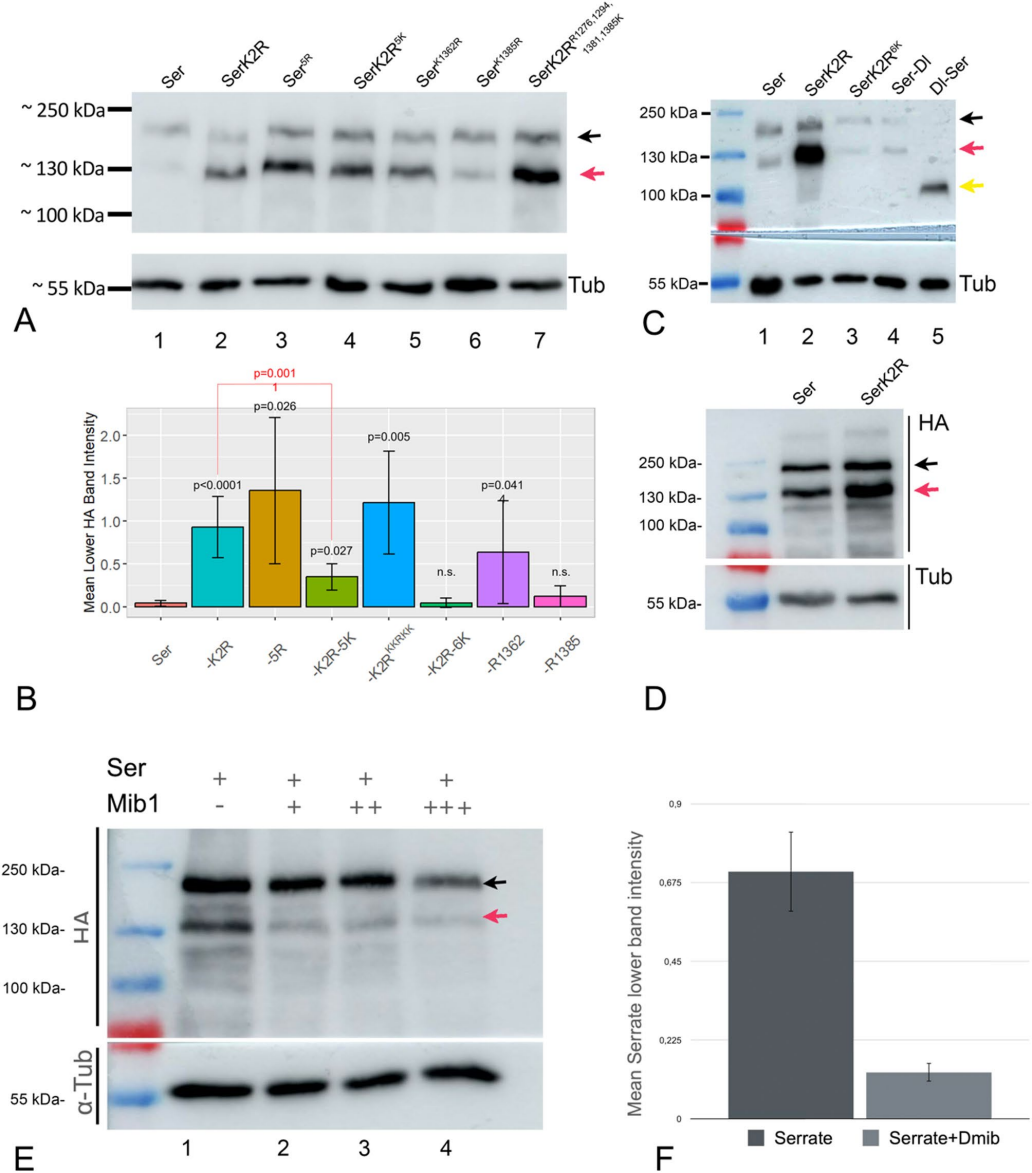
Western Blot analysis of Ser variants, detected by an antibody directed against the HA tag,. **A** Ser runs in two bands in the WB (black and red arrow). Note, the dramatic increase in the intensity of the FMB in the case of SerK2R compared to Ser (Lane 1 and 2, red arrow). **B** Quantification of the intensity of the FMB (lower band) normalized to the Tubulin loading control (n=5, HA staining as in (**A**)). **C** Western-Blot analysis of additional Ser-variants, including SerK2R^6K^. Note, that the intensity of the FMB is reduced to the level of Ser, suggesting that the additional introduction of K1370 also restores bulk endocytosis (lane 3, compare with lane 1, quantification in **B**). **D** Western-Blot analysis of the expression of Ser and SerK2R in S2R+ cells. **E**, **F** WB analysing the expression of Ser in S2R+ cells in the absence (lane 1) and presence of increasing amount of Mib1 (lanes 2–4). Like in the disc cells, two main bands of Ser are present (arrows). Note, that the intensity of the FMB (red arrow) decreases with increasing amount of Mib1, suggesting that the corresponding fraction of Ser undergoes Mib1-mediated degradation. **F** Quantification of the intensity of the FMB of the Blot shown in (**D**), normalised to the Tubulin control. The experiment is repeated three times (n=3) with the 1: 3 relation of Ser: Mib1 (lane 4 in D)

To further investigate the endocytic behaviour of our Ser variants, we performed an antibody uptake assay in *Drosophila* S2R + cells developed by Glittenberg et al. [15]. In these experiments, Ser or SerK2R were expressed in the presence or absence of Mib1. The fraction of Ser at the plasma membrane was labelled by incubation of living cells with an anti-Ser antibody directed to the ECD, initially at 4 °C to prevent endocytosis during the time of incubation (outlined in Fig. 3G). The antibody-labelled membrane fraction of Ser was chased through the endosomal pathway after shifting the temperature to endocytosis permissive 25°C. At chase time 0´, the antibody-labelled Ser was present at the cell surface, confirming the lack of endocytosis at 4 °C (Fig. 3H, H’ I, I’, J, J’, K, K’). In the absence of Mib1, neither Ser nor SerK2R were efficiently internalized into the cells, even after a chase of 60´ (Fig. 3H1-H2’, J-J2’, quantification in N). This suggests that no E3-ligase relevant for Ser endocytosis is expressed at a significant level in S2R + cells (see also below Fig. 7D, E). In contrast upon co-expression with Mib1, the Ser-signal disappeared from the cell surface and was only detectable in Rab7-positive endosomes already after 30 min. (Fig. 3I-I2’, quantification in N). The location in endosomes was still detectable after a 60 min. chase. These observations confirm similar previously performed experiments that Mib1 can induce the rapid endocytosis of Ser from the cell surface [15]. In contrast to Ser, SerK2R was still present in the plasma membrane even after a chase of 60 min. although Mib1 was co-expressed (Fig. 3K-K2’, quantification in N). These results further confirm the requirement for the presence of the Ks in the ICD for the efficient endocytosis of Ser by Mib1.

Most of the ubiquitylated endocytosed cargo is transported via the endosomal pathway to the lysosome to be degraded. To show that the Ks and ubi are also necessary for the degradation of Ser, we performed pulse-chase experiments in the wing imaginal disc, as we have previously performed with Dl [8]. Using the TARGET-system, we expressed Ser and SerK2R for 16 h in the anterior compartment of the wing disc by a combination of *ci*Gal4 and *tub*Gal80^ts^. After the 16 h pulse of expression, we changed to the restrictive temperature to halt the expression and chase the HA signal through increasing time intervals (Fig. 4A). We found that Ser-HA expression vanished completely in the interval between 12 h and 24 h (Fig. 4B-B’’’). In contrast, SerK2R could be detected in the apical membrane of disc cells, even after a chase of 72 h (Fig. 4C-C’’’, yellow arrow). This indicates that SerK2R is much less efficiently degraded and endocytosed and further supports the notion that Ser requires its Ks for its efficient degradation.

### Identification of Ks in the ICD of Ser relevant for its function

E3-ligases can ubiquitylate their substrates rather unspecific, or selectively at specific Ks. To identify Ks in the ICD of Ser which are important for Mib1-dependent activation, we focussed on the five highly conserved Ks (core Ks, Fig. S4A and Fig. S1A). We performed two sets of complementary experiments to determine their meaning: We first replaced the Ks by Rs or re-introduced them into SerK2R. We then tested the activity of these variants in our assay. A complete summary of our results is shown in table S1 and in Fig. S4. Here, we highlight the important findings. To determine the signalling activities of the variants, the lengths of anterior and/or posterior stripe of ectopic Wg expression were measured, summed up and related to the length of the endogenous Wg expression along the D/V-boundary for normalization. CI was quantified as a percentage of the length of the gap in the endogenous expression of Wg relative to its length (for further information, see Methods). The dominant-negative acting variants were excluded from analysis, since it is not clear whether the underlying mechanism is the same as in the case of CI.

The replacement of all five core Ks by Rs in the ICD (Ser^K1276,1294,1362,1381,1385R^, from hereon Ser^**5R**^) caused a dominant-negative phenotype that is indistinguishable from that of SerK2R, indicating that it acts in a dominant-negative manner (Fig. 5A, red bracket, compare with Fig. 2D’). This is further confirmed by our finding that, just like SerK2R, Ser^**5R**^ cannot induce ectopic Wg expression, even in co-expression with Notch (Fig. 5B, compare with Fig. 2E). However, endocytosis of Ser^**5R**^ was observed, indicated by the HA-positive punctae in the expressing cells (Fig. 5A’, arrowheads). Moreover, in contrast to SerK2R, the signalling activities of Ser^**5R**^ were slightly re-established by co-expression of Mib1, indicating that additional K(s) might be marginally involved in Mib1 signalling in addition to the core Ks (Fig. 5C. C’). Signalling-inactivity was also observed for Ser-variants where four of the five core Ks were absent (Fig. S4R-V). These findings suggest that the less conserved Ks in the ICD cannot really compensate for the loss of four of the five core Ks.

As expected, the individual re-introduction of only one core K into SerK2R was not sufficient to restore the signalling activity of SerK2R, indicating that more than one K is required for the activity of Ser (Fig. 5E, red bracket and Fig. S5D-G, compare with Fig. 2D, D’). Expression of the SerK2R-variant where all five core Ks were re-introduced (SerK2R^5K^), caused a phenotype that was similar to that of Ser (Fig. 5D, white arrows, compare with Fig. 2B, quantification in K). Interestingly the anterior stripe of ectopic Wg expression was longer and better defined, suggesting an increase in signalling, compared to Ser. This finding was confirmed by the quantification of signalling, shown in (Fig. 5K). Altogether, the results indicate that the 5 most conserved Ks are crucial and sufficient for the signalling activity of Ser.

Next, we explored the importance of individual core Ks. The individual replacement of the core Ks by R had different effects on the activity of Ser (Fig. 5F-H; Fig. S5P, Q). The individual replacement of K1276, K1381 or K1385 by R lead to the loss of the anterior stripe of ectopic Wg expression (Fig. 5F, yellow arrow, quantification in K, Fig. S4P, Q). The replacement of only K1294 (Ser^K1294R^) had a slight effect on signalling, but did not affect the cis-inhibitory ability of Ser (Fig. 5G, white arrow, quantification in K). Nevertheless, the effects of the replacement of the Ks were generally weak in comparison to the replacement of K1362, which caused a very strong loss of function phenotype. Expression of Ser^K1362R^ induced none, or only very minor ectopic Wg expression restricted to the D/V-region in a fraction of discs (Fig. 5H, white arrow, quantification in K). In addition, it caused a larger gap than Ser in the endogenous expression of Wg along the D/V-boundary in a cell-autonomous manner (Fig. 5H, yellow arrow and bracket, quantification in K, compare with Fig. 2B). This observation indicates that Ser^K1362R^ has lost most of the signalling activity. The cell-autonomous interruption of Wg expression indicates that, in contrast to SerK2R, Ser^K1362R^ is not a dominant-negative variant of Ser (Fig. 5H, white arrow). In support of this conclusion, we found that its co-expression with Notch, or with Mib1, resulted in a strong ectopic activation of Wg (Fig. 5I, J, arrows). In the discs only few intracellular HA-positive punctae are observed and we observed accumulation of Ser^K1362R^ in the basal membrane of the epithelium, as observed for SerK2R (Fig. 5H, H’’, red arrow, compare with Fig. 3C’). This suggests that Ser^K1362R^ is less efficiently endocytosed. This conclusion is further supported by the finding that Mib1-mediated ubi of Ser^K1362R^ was strongly impaired in the Ubi-assay in S2+-cells (Fig. S3B, lane 6).

To further reveal the reduced endocytosis of Ser^K1362R^, we performed the described antibody uptake experiment in S2R + cells (Fig. 3L-M2’). Ser^K1362R^ could be internalised into the S2R + cells when co-expressed with Mib1. However, this internalisation was less efficient compared to that of Ser, but faster than SerK2R (see quantification in N). These results indicate that K1362 is the most important K for signalling and ubi-induced endocytosis of Ser.

### SerK2R and Ser^K1362R^ physically interact with Mib1

Previous work showed that binding of Mib1 to the ICDs of the ligands is required for signalling [16, 39]. Thus, a simple reason why Mib1 cannot induce the endocytosis and activity of SerK2R and Ser^K1362R^ is that it is unable to bind these variants. To exclude this possibility, we perform co-immunoprecipitation experiments. We co-expressed Ser, SerK2R or Ser^K1362R^ with V5-Mib1ΔRING in S2R + cells. Mib1ΔRING, which lacks the catalytical RING domain, has been shown to more efficiently co-immunoprecipitate with the ICD of Dl and Ser than full-length Mib1 [39]. We found that Ser, SerK2R and Ser^K1362R^ co-precipitated with V5-Mib1ΔRING, ruling out the possibility that Mib1 is not able to bind these variants (Fig. 5L). The detected physical interaction of Mib1 with Ser^K1362R^ is in agreements with of our finding that Ser^K1362^ becomes strongly active if co-expressed with Mib1 in the wing imaginal disc (Fig. 5J, arrows).

### The presence of all core Ks is required for the full activation of Ser by Mib1

With exception of K1362, the individual replacement of none of the other conserved Ks in the ICD had a strong effect on the activity of Ser. This includes K1276 and K1294 which have been reported to be very important for the activity of Ser, based on their replacement by alanine (A) [15]. However, the replacement by the structurally different A might affect the conformation of the Ser-ICD. We therefore replaced both Ks by Rs. We found that Ser^K1276,1294R^ had considerable more activity than reported for the replacement of the Ks by A [15] (Fig. 6A, B, arrow in B, quantification in J). Nevertheless, the activity of Ser^K1276,1294R^ was significantly weaker than that of Ser, indicated by the reduction in the expression of ectopic Wg expression (Fig. 6B compare with Fig. 2, B’, white arrow, quantification in J). It also had slightly increased cis-inhibitory abilities (Fig. 6B, yellow bracket, compare with Fig. 2B, B’, quantification in J). The finding confirms that the two Ks contribute to the activity of Ser (signalling and CI), but also indicates that more than two core Ks are required for its full activity. In addition, the difference in the outcome caused by the replacement of the Ks by alanine or arginine, suggests that the side chain and/or charge at position 1276 and 1294 is important for the function of Ser. It is therefore likely that K1276 and K1294 have an additional structural importance besides its function as ubi acceptors. Importantly, even SerK2R variants where four of the five core Ks were re-introduced were only weakly active, or even inactive (Fig. 6D-F; Fig. S4L, M): the individual replacement of either K1276, or K1294, or K1381 from SerK2R^**5K**^ by R led to a dramatic reduction in activity (Fig. 6D, E, white and yellow arrow, quantification in J; Fig. S4M), while the variants without K1385 (SerK2R^R1276,1294,1362,1381 K^, F) or K1362 (SerK2R^R1276,1294,1381,1385 K^) displayed even a dominant-negative behaviour (Fig. 6F, red bracket; Fig. S4L). In summary, the experiments indicated that the presence of all five core Ks is required for the full activity of Ser. They further reveal a hierarchy in the importance with K1362 being the most important followed by K1385 and then the other three core Ks.

We initially attempted to generate knock-ins of Ser-ICD-variants into *Dl*^*attP*^ as described for *Dl*^*attP*^*-Dl-Ser*. However, we failed to obtain flies carrying a *Dl*^*attP*^*-Dl-SerK2R* allele, although we tried for several times. We suspect that the strong dominant-negative activity of the SerK2R-ICD makes the generation of transformants impossible. Nevertheless, we succeeded to generate an *Dl*^*attP*^*-Dl-SerK1362R* allele. Flies heterozygous for this allele displayed a dominant phenotype in the wing, similar to *Dl* heterozygous flies, (Fig. S1D, arrows). In addition, the expression of the Notch activity marker Gbe + Su(H) was reduced in these *Dl*^*attP*^*-Dl-SerK1362R/+* flies, indicating a dominant negative effect on Notch activity (Fig. S1I’’, white and red arrow, compare with G’’). *Dl*^*attP*^*-Dl-SerK1362R/Df flies*, where only Dl-SerK1362R is present, die during development with escapers that develop to the pharate adult stage. These escapers displayed strong leg defects, which are typical for *Dl*-mutants (Fig. S1F) [40, 41]. Moreover, *Dl*^*attP*^*-Dl-SerK1362R/Dl*^*attP*^*-Dl-Ser* flies displayed a strong leg and wing phenotype that was stronger than flies carrying the two alleles in heterozygousity (Fig. S1C-D’). These experiments show that the exchange of K1362 led to a severe reduction of the function of the Ser-ICD, as we have observed in the over-expression experiments. They also confirm that K1362 is very important for the function of the ICD of Ser. The clonal analysis revealed that homozygousity of *Dl*^*attP*^*-Dl-SerK1362R* is cell lethal for wing disc cells, indicated by the sole presence of orphan wildtype clones (Fig. S1J, J’, arrows). The cell lethality supports the notion that the replacement of K1362 in Dl-Ser results in a strong loss of function. Interestingly, K1362 is located at the N-terminal side of the predicted CB of Ser, a motif that required for efficient binding of MIB1 to JAG1 (Fig. S2E) [16]. However, the functionality of the predicted C-box of Ser has not been validated.

### A restricted flexibility in the use of Ks for Ser-signalling

The results indicated that the presence of all five core Ks is required for the signalling activity of Ser. The loss of each of them in the ICD of SerK2R^**5K**^ led to complete loss or strong reduction in activity in Mib1-dependent activation. However, the complementary analysis with Ser also showed that the individual loss of the core Ks had minor effects on function, with the exception of K1362 (see Fig. 5D-E; Fig. S5P, Q). This suggests that other, less conserved Ks of the ICD of Ser can compensate for the individual loss of some core Ks. To test this conclusion, we asked whether the re-introduction of K1370, which is slightly less conserved, leads to a gain of activity of two SerK2R^**4K**^ variants, SerK2R^R1294,1362,1381,1385 K^ and SerK2R^R1276,1294,1362,1381 K^, which displayed very weak or no activity, respectively (Fig. 6D, F). We found that the additional re-introduction of K1370 into these 4 K-variants increased the level of activity of these variants close to the wildtype: Expression of SerK2R^R1294,1362,**1370**,1381,1385 K^ or SerK2R^R1276,1294,1362,**1370**,1381 K^ induced a robust ectopic stripe of Wg expression in posterior boundary cells (Fig. 6G, white arrow, quantification in J; Fig. S4O). Thus, the addition of K1370 can partially compensate for the loss of one core K.

To further investigate the issue of selectivity in the use of the Ks, we also re-introduced K1349 to the 4 K variants, which is similarly well-conserved as K1370 (Fig. S2A). The resulting variants, SerK2R^R1294,**1349**,1362,1381,1385 K^ and SerK2R^R1276,1294,**1349**,1362,1381 K^, also showed an increase in activity. However, the increase was smaller than in the case where K1370 were introduced (Fig. 6H compare E, white arrow, quantification in J; Fig. S4N). This indicates that K1370 and K1349 can compensate for the loss of a core K to a different extent. Thus, there is a certain flexibility of Mib1 in the use of the Ks. This flexibility explains why the individual loss of the core Ks does not strongly affect the activity of Ser, with the exception of K1362.

Finally, we also introduced K1370 into SerK2R^**5K**^ to generate a SerK2R^**6K**^ (SerK2R^R1276,1294,1362,**1370**,1381,1385 K^). We asked whether six Ks in the ICD of SerK2R might further improve the activity of the ligand. SerK2R^**6K**^ did not show significantly more signalling activity compared to SerK2R^**5K**^, indicating that the five core Ks are sufficient for the Epsin-dependent signalling function of Ser (Fig. 6I compare with Fig. 5C, white arrow, quantification in J).

### Western-Blot analysis reveals differences in the degradation of the Ser-variants

To monitor the protein level of the Ser-variants, we performed Western-Blot (WB) analysis (Fig. 7). For this analysis, a selection of the variants was ubiquitously expressed for 24 h using a combination of *tub*Gal4 with *tub*Gal80^ts^ in wing imaginal discs (TARGET system) [42]. This led to a robust ubiquitous expression in disc cells, without detectable morphological consequences or apoptosis. Note, that all constructs are inserted in the same genomic landing site to guarantee comparable transcription levels.

In the WB, all Ser-variants migrated in the previously described two band pattern: a slower and a faster migrating band (SMB and FMB, respectively; Fig. 7A, lane 1, black and red arrow) [43]. The occurrence of two differentially migrating Ser-species indicates that Ser is probably modified. We found that this modification occurs in the ECD, as it is not observed in a hybrid ligand, consisting of the ECD of Dl and the ICD of Ser (Dl-Ser-HA), but was present in the complementary hybrid Ser-Dl-HA ligand (Fig. 7C, lane 4 and 5). Expression of Ser and SerK2R in S2 + cells resulted in an intensity of the of the FMB of SerK2R compared to Ser, suggesting that the FMB is stabilised in the absence of Mib1 (Fig. 7D, black and red arrow).

Importantly, the WB revealed consistent differences in the degradation of the analysed variants: We found that the intensity of the FMB varied strongly among the variants (Fig. 7A, quantification in B): In the case of Ser, the intensity of the FMB was low compared to the SMB, indicating that it comprises a small percentage of the total amount of Ser present in wildtype cells (Fig. 7A, lane 1, quantification in B). In contrast, in the case of SerK2R, the intensity of the FMB was dramatically increased and even stronger than the SMB (Fig. 7A, lane 2, quantification in B). Likewise, the FMB of Ser^5R^, where the core Ks are replaced, is also dramatically increased (Fig. 7A, lane 3, quantification in B). The increase suggests that the FMB contains the fraction of Ser that is subjected to ubi by E3-ligases and subsequent degradation. Consequently, it is much weaker in the case of Ser compared to SerK2R and Ser^5R^. To further support this notion, we expressed Ser with and without Mib1 in S2 + cells. We detected the FMB and SMB of Ser in WB of S2 + cells expressing Ser (Fig. 7E, red and black arrow). The intensity of both bands was similar in the absence of Mib1 (Fig. 7E, D, lane 1). In contrast, the intensity of the FMB was dramatically reduced upon co-expression of Ser with Mib1. The reduction of the intensity of the FMB was dependent on the concentration of Mib1 (Fig. 7E, lane 2–4, quantification in F). Moreover, in the case of expression of SerK2R, the intensity of the bands was increased compared to Ser, suggesting a greater stability, especially of the FMB (Fig. 7E, red arrow). Altogether, these results indicate that the presence of Mib1 preferentially induces the rapid endocytosis and subsequent degradation of the fraction represented by the FMB. Hence, the intensity of the FMB is a measure for the efficiency of a given variant to be degraded and, indirectly, also for its endocytosis behaviour.

The quantification of the WB analysis of expression of the Ser-variants in discs revealed that the intensity of the FMB of Ser^K1362R^ is higher than that of Ser, confirming less efficient degradation/endocytosis (Fig. 7A, lane 5, quantification in B). Likewise, a non-functional SerK2R-variant where only four of the core Ks re-introduced (*SerK2R*^*R1294,1362,1381,1385 K*^), displayed a more intense FMB, indicating a reduction in degradation/endocytosis (Fig. 7A, lane7, quantification in B). Interestingly, the intensity of the FMB of SerK2R^**5K**^ is higher than that of Ser (Fig. 7A, lane 4, quantification in B). Thus, although even slightly more active than Ser, SerK2R^**5K**^ appears to be less efficiently endocytosed/degraded. This finding is compatible with the notion that the presence of only the 5 core Ks selectively restores the signalling-relevant endocytic pathway. Importantly, the further addition of K1370 (SerK2R^6K^) leads to the reduction of intensity of the FMB to a level comparable to Ser (Fig. 7C, quantification in B). Since SerK2R^6K^ is similarly active than SerK2R^5K^, the finding suggests that the addition of one additional K restores also the signalling-irrelevant bulk endocytosis of Ser in addition to the signalling-relevant Epsin-dependent pathway. Thus, 6 Ks of the ICD appear to be required for the complete endocytic behaviour of Ser. Altogether the WB analysis revealed quantitative differences in the degradation/endocytosis of the variants, dependent on the presence of the 6 most conserved Ks. These differences correlate with their signalling behaviours and endocytosis behaviour in wing imaginal disc cells. Variants with a high intensity of the FMB tend to have a reduced signalling activity and variants with a low intensity of the FMB have a similar or even slightly higher signalling activity than Ser.

## Discussion

Previous over-expression experiments with Dl and Ser revealed differences in their ability to activate Notch signalling. For example, Li and Baker [32] reported that Ser is more cis-inhibitory than Dl and that the ECD of the ligands is responsible for this difference [32]. Our analysis of the *Dl*^*attP*^*-Dl-Ser* knock-in allele indicates that also their ICDs contribute to the differences between the ligands. One important difference is that the ICD of Ser mediates higher CI than the Dl-ICD. The higher CI correlates with less efficient endocytosis and a dominance in signalling compared to Dl-expressing cells. The inverse correlation between endocytosis efficiency and CI was recently also reported for the ICD of Dl [14]. Our work supports the conclusion of previous work about the correlation between endocytosis and signalling of Ser [15]. It appears that the endocytosis efficiency of a given ICD regulates the amount of a ligand at the plasma membrane and thereby determines the amount of Notch engaged in cis- and trans-interaction with it. In our clonal analysis, Dl-Ser was not only more efficient in CI, but also dominant in signalling compared to Dl. We believe that the higher concentration of Dl-Ser on the cell surface results also in a higher likelihood to trans-interact with Notch displayed by adjacent Dl-expressing cells. In the adjacent homozygous Dl clone, less Dl is at the surface of cells, because the ICD of Dl mediates more efficient endocytosis. Therefore, less Notch is engaged in CI and consequently available for the trans-interaction with Dl-Ser. Moreover, the high level of Dl-Ser in the Dl-Ser homozygous clone can efficiently compete with cis-interacting Dl for binding to Notch in the adjacent cells. Thus, the differences in the endocytosis rate translate into differences in signalling, making Dl-Ser expressing cells better signallers and worse receivers than Dl expressing cells. What properties contribute to the differences in the ability of the two ICDs to mediate endocytosis? We found that the Ser-ICD is not able to mediate the ubi- and Mib1-independent endocytosis activity, which is observed for the Dl-ICD [8, 14]. It is likely that this additional mode of endocytosis contributes to the higher endocytosis efficiency of the Dl-ICD and therefore to the reduced presence of the connected ligand at the plasma membrane compared to the ICD of Ser. It is important to determine why only the ICD of Dl can signal via ubi-independent modes in future experiments. Sequence comparisons and modelling the structures with Alphafold2 did not reveal any obvious differences, despite the fact that the ICD of Ser is shorter. Both ICDs are predicted to be intrinsically disordered. A thorough structure function analysis is required to identify the regions responsible for the ubi-independent signalling mediated by the ICD of Dl.

In the previous analysis of Dl, we found that the Ks of its ICD are crucial for the suppression and therefore adjustment of CI, as well as its signalling ability [8, 14]. This motivated us to monitor the meaning of the Ks and ubi for the activity of Ser in Mib1-mediated, ligand-dependent Notch activation. We found significant differences in the importance of the Ks for Ser in comparison to Dl. Unlike DlK2R, SerK2R is inactive and behaves in a dominant-negative fashion, just like Dl- and Ser-variants without an ICD, highlighting the importance of the Ks for the activity of Ser. The behaviour of SerK2R in wildtype cells resembled that of Ser in *mib1* mutant cells with respect to both endocytosis and signalling and its signalling activity cannot be resurrected by co-expression of Notch or Mib1. These observations indicate that the Ks of Ser are essential for Mib1-mediated Ser-signalling. Nevertheless, the two ligands share similarities in the meaning of the Ks of their ICDs. In both cases, half of the Ks of the ICDs are important for function, in the case of Dl 6 out of 12 Ks and in the case of Ser 5 out of 10 Ks [5]. In both ICDs, there is a clear correlation between the presence of the most conserved core Ks, endocytosis and signalling and an inverse correlation between endocytosis and CI [8, 14]. Thus, it appears to be a general rule that the Ks in the ICD dictating the endocytosis behaviour and signalling activity of a given ligand of the Notch pathway through their arrangement and number. In this way, they also determine its cis-inhibitory abilities. Another interesting similarity between Dl and Ser is the outstanding importance of one K in the C-terminus of their ICD. In the case of Dl, our previous work revealed that K762 is by far the most important K for signalling [5]. Likewise, K1362 is by far the most important one for signalling of Ser. In the case of Dl, K762 is located at the N-terminus of its functional C-box, which is one epitope of the bipartite binding site of Mib1 [5, 9, 16] (Fig. S2E). Similarly, K1362 is at the N-terminus of the predicted CB in Ser (Fig. S2E). It will be interesting to investigate the reason behind the singular importance of these Ks in the future. One interesting possibility is that ubi of K1362 leads to a structural change in the ICD that increases the efficiency ubi of core Ks at other positions. Besides showing the importance for signalling of Ser, our work also hints to a possible suppressive function of some unidentified Ks: Our results show that the SerK2R^5K^ and SerK2R^6K^ are more potent to activate the Notch pathway than Ser, indicated by the increased length of the anterior stripe of ectopic expression of Wg. In the future, it will be interesting to identify these Ks and their function.

Previous work indicates that two different endocytosis pathways account for the endocytosis and degradation of Dl and Ser (reviewed in [1]). Bulk endocytosis is responsible for the majority of ligand endocytosis, whereas Epsin-dependent endocytosis is signalling relevant, but accounts only for a small fraction. In the case of Ser, the loss of *mib1* function abolishes endocytosis completely, indicating that both endocytosis pathways are initiated via ubi by Mib1. In the case of Dl, bulk endocytosis is independent of Mib1 and might be initiated by another unidentified E3-ligase [14]. We found that the five most conserved Ks of Ser orthologs among insect species, are essential for the signalling activity of Ser. The combined loss of these core Ks resulted in a phenotype that resembled that of SerK2R, despite the presence of five remaining Ks. Hence, losing the core Ks renders the ICD functionless for Mib1-mediated activation of Ser. Interestingly, the Western-Blot analysis combined with the in vivo analysis revealed that the endocytosis of SerK2R^5K^ was strongly reduced compared to Ser, although it possessed the full signalling abilities. It has been previously shown that signalling of Ser depends on the activity of *lqf*, implicating that ubi of the core Ks is recognised by Epsin [17]. We therefore conclude that ubi of the core Ks by Mib1 preferentially channels Ser into the Epsin-dependent endocytosis pathway, but it is not sufficient for prevailing efficient bulk endocytosis. The finding that Ser^5R^, which has lost its signalling capacity, is also not efficiently endocytosed, suggests that the core Ks are also involved in bulk endocytosis. Hence, bulk endocytosis must be mediated by a combination of the core Ks with some of the remaining 5 Ks. We have identified K1370 as one link, as its addition to the core Ks can re-install the complete endocytosis behaviour of Ser. The results combined with previous ones suggest that for bulk endocytosis, Ser must be linked to the endocytic core machinery via an endocytic adapter other than Epsin, since it has been shown that loss of *lqf* function does not grossly affect the endocytosis of Dl and Ser in wing disc cells [17]. In contrast to Epsin/Lqf, this unidentified adapter-mediated endocytosis appears not be able to generate the required ligand-pulling force to activate Notch. Nevertheless, the evidence so far suggest that it must be able to bind to ubiquitin, as Mib1 and Ks are required for bulk endocytosis of Ser. One obvious candidate is Eps15, which is the only other adapter known to contain UIMs. It can physically interact with Epsin and the core endocytic machinery via AP-2, but lacks two properties of Epsin: it lacks the membrane curvature inducing ENTH domain and cannot not bind to and organise Actin filaments. Thus, in contrast to Epsin, it is probably not able to generate the pulling force necessary for activating Notch. Another outstanding question that has to be addressed is how the entry into the two pathways is regulated. Both adapters are ubiquitously expressed, raising the possibility that their competition for ligand binding determines the fractions of ubiquitylated Ser entering a particular pathway and thereby determines the level of signalling. Epsin and Eps15 can physically interact [44]. Thus, it is alternatively possible that only the endocytic pits containing both UIM possessing adaptors that produce sufficient pulling force on the ligand during endocytosis to activate the Notch pathway. Both adapters possess two UIMs and this might explain why multiple Ks are required for the proper activation of signalling of Ser and why the reduction of a single core K has such a great reducing effect on signalling of SerK2R^5K^. However, recent work also suggests that the trans-interaction between the ligand and Notch contributes to the decision to enter the Epsin-dependent pathway [45]. The interplay between Epsin and Eps15 might also lead to an explanation for the puzzling observation that the five core Ks are sufficient for the re-establishment of signalling, but not for the re-establishment of the complete endocytic behaviour of Ser. Future work should address and clarify these questions.

## Conclusions

Our work revealed significant differences in the properties of the intracellular domains of the two founding members of the DSL-family of Notch ligands. It further indicates that selective ubiquitylation of 5 conserved lysins in the ICD by the E3-ligase Mib1 is required for signalling of Ser.

## Methods

### Drosophila stocks and genetics

All fly experiments were performed at 25 °C, unless indicated otherwise. For experiments with tubGAL80ts, the flies were kept at 18–21 °C and then shifted to 29 °C for the desired time.

**Table:** Fly stocks used in this study

Genotype	Source or Reference
Gbe + Su(H)-*lacZ*	Furriols, M. Bray, S.
*ptc*GAL4, UAS-*GFP*	Speicher et al. 1994 [31] (ptc) Yeh et al.1995 (GFP)
*ci*Gal4 *tub*Gal80^ts^	Croker et al. 2006, BDSC 7108
*tub*Gal4 *tub*Gal80^ts^	BDSC_5138; BDSC_7018
*mib1* ^*EY09870*^	Lai et al., 2005 [39]
UAS-*N (III)*	Klein and Martinez-Arias, 1998
*ptc*GAL4, *Lqf*^*FL*^-GFP	Xie et al. 2012 [12]; this study
*Dl* ^*BSC850*^	BSC27922
*Dl-* ^*attP*^ *-Dl-HA*	[14]
*Dl* ^*attP*^ *-Ser-HA*	this study
UAS-*Ser-HA*	Berndt et al.
UAS-*Ser*^*K1276,1294,1362,1381,1385R*^*-HA* (*Ser*^*5R*^*-HA*)	this study
UAS*-Ser*^*K1276R*^*-HA*	this study
UAS*-Ser*^*K1294R*^*-HA*	this study
UAS-*Ser*^*K1362R*^*-HA*	this study
UAS*-Ser*^*K1381R*^*-HA*	this study
UAS-*Ser*^*K1385R*^*-HA*	this study
UAS*-Ser*^*K1276,1294R*^*-HA*	this study
UAS*-Ser*^*K1362,1381,1385R*^*-HA*	this study
UAS-*Ser*^*K1294,1362,1381,1385R*^*-HA*	this study
UAS-*Ser*^*K1276,1362,1381,1385R*^*-HA*	this study
UAS-*Ser*^*K1276,1294,1381,1385R*^*-HA*	this study
UAS-*Ser*^*K1276,1294,1362,1385R*^*-HA*	this study
UAS-*Ser*^*K1276,1294,1362,1381R*^*-HA*	this study
UAS-*SerK2R-HA*	this study
UAS-*SerK2R*^*R1276K*^*-HA*	this study
UAS-*SerK2R*^*R1294K*^*-HA*	this study
UAS-*SerK2R*^*R1362K*^*-HA*	this study
UAS-*SerK2R*^*R1381K*^*-HA*	this study
UAS-*SerK2R*^*R1385K*^*-HA*	this study
UAS-*SerK2R*^*R1276,1294 K*^*-HA*	this study
UAS-*SerK2R*^*R1276, 1294,1362 K*^*-HA*	this study
UAS-*SerK2R*^*R1276,1294,1385 K*^*-HA*	this study
UAS-*SerK2R*^*R1362,1381,1385 K*^*-HA*	this study
UAS-*SerK2R*^*R1294,1362,1381,1385 K*^*-HA*	this study
UAS-*SerK2R*^*R1276,1362,1381,1385 K*^*-HA*	this study
UAS-*SerK2R*^*R1276,1294,1381,1385 K*^*-HA*	this study
UAS-*SerK2R*^*R1276,1294,1362,1385 K*^*-HA*	this study
UAS-*SerK2R*^*R1276,1294,1362,1381 K*^*-HA*	this study
UAS-*SerK2R*^*R1276,1294,1362,1381,1385 K*^*-HA* (UAS-*SerK2R*^*5K*^*-HA*)	this study
UAS-*SerK2R*^*R1294,1362,1370,1381,1385 K*^*-HA*	this study
UAS-*SerK2R*^*R1276,1294,1362,1370,1381 K*^*-HA*	this study
UAS-*SerK2R*^*R1294,1349,1362,1381,1385 K*^*-HA*	this study
UAS-*SerK2R*^*R1276,1294,1349,1362,1381 K*^*-HA*	this study
UAS-*SerK2R*^*R1276,1294,1362,1370,1381,1385 K*^*-HA* (UAS-*SerK2R*^*6K*^*-HA*)	this study
UAS-*Ser*^*RQRL*^*-HA*	this study

### Generation of the constructs

SerK2R-HA was generated by replacing the ICD of Ser-HA with a synthesised ICD in which all Ks replaced by Rs. The synthesis of K2R-ICD was performed by GenScript. Ser-HA and SerK2R-HA were subcloned in the pBluescript and used as templates for site directed mutagenesis (SDM) to generate different Ser-Rs and SerK2R-Ks variants. The oligonucleotides used are listed in the table. After the SDM, the constructs were cloned back into the origin vectors pUAST attB-Ser-HA and pUAST attB-SerK2R-HA via the cleavage sites AatII + XhoI and AatII + NotI, respectively. All constructs were sequenced before injection and inserted into the landing site attP51C.

**Table:** List of oligonucleotides used in this study

Primer name	Sequenz 5´◊3´
Ser-K1276R_for:	GCGTCACGAGGAGGAGCGGTCGAATAATCTGCAG
Ser-K1276R_rev	CTGCAGATTATTCGACCGCTCCTCCTCGTGACGC
Ser-K1294R_for	GTATACAAATCCGCTGCGGGGCAGCACCAGTTCC
Ser-K1294R_rev	GGAACTGGTGCTGCCCCGCAGCGGATTTGTATAC
Ser-K1362R_for	CACAGATTCTGCTGCACCGAACCCAAAACTCGGAC
Ser-K1362R_rev	GTCCGAGTTTTGGGTTCGGTGCAGCAGAATCTGTG
Ser-K1381R_for:	GACAGTCCGCGTCGGGACTTTGGCAAG
Ser-K1381R_rev:	CTTGCCAAAGTCCCGACGCGGACTGTC
Ser-K1385R_for:	GTAAGGACTTTGGCCGGCGGTCGATCAACTG
Ser-K1385R_rev:	CAGTTGATCGACCGCCGGCCAAAGTCCTTAC
Ser-K1381,1385R_for	CTGGACAGTCCGCGTAGGGACTTTGGCAGGCGGTCGATCAACTG
Ser-K1381,1385R_rev	CAGTTGATCGACCGCCTGCCAAAGTCCCTACGCGGACTGTCCAG
SerK2R-R1362K_for	GCTCACAGATTCTGCTGCACAAAACCCAAAACTCGGACATGCG
SerK2R-R1362K_rev	CGCATGTCCGAGTTTTGGGTTTTGTGCAGCAGAATCTGTGAGC
SerK2R-R1381K_for	CTGGACAGTCCGCGTAAGGACTTTGGCCGGCG
SerK2R-R1381K_rev	CGCCGGCCAAAGTCCTTACGCGGACTGTCCAG
SerK2R-R1385K_for	CGTCGGGACTTTGGCAAGCGGTCGATCAACTG
SerK2R-R1385K_rev	CAGTTGATCGACCGCTTGCCAAAGTCCCGACG
SerK2R-R1370K_for	CAAAACTCGGACATGCGGAAGAACACTGTGGGCTCGC
SerK2R-R1370K_rev	GCGAGCCCACAGTGTTGCGCCGCATGTCCGAGTTTTG
SerK2R-R1349K_for	TCCAGTACGGGCCTGAAGCAGGCGCACCGCCGGAGC
SerK2R-R1349K_rev	GCTCCGGCGGTGCGCCTGCTTCAGGCCCGTACTGGA
Ser-ICD_RQRL-for	ATCAGTCTTTACTGGAGACAGCTCTGTACCCATACGACGTT
Ser-ICD_RQRL-rev	AACGTCGTATGGGTACAGACGCTGTCTCCAGTAAAGACTGAT

While working on this manuscript, we noticed that the amino acid sequence of the Ser variant we use differs slightly from the Ser variant published in c. (2006). Our variant has an insertion of four amino acids and a replacement of A by P, respectively in the signal peptide sequence (see alignment, green arrows). The difference in the sequence has no effect on the function of the ligand. However, it is the reason why our numbering of Ks in the ICD deviates from that of Glittenberg et al. [15]. Thus, K1276 and K1294 correspond to K1269 and K1287 in Glittenberg et al. [15].


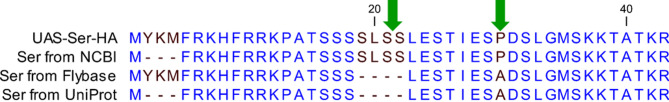


### S2R + cell culture

S2R + cells (DGRC # 150) were grown in Schneider´s medium (Pan Biotech) with 10% FBS (P30-1502, Pan Biotech) and 1% penicillin-streptomycin (Invitrogen) at 25 °C. The S2R + cells were transfected at a confluence of approx. 50% using TransIT-Insect Transfection Reagent from Mirus according to the manufacturer’s instructions. 600–700 ng of plasmid DNA were transfected. After transfection, the cells were incubated for 48 h at 25 °C. Ser constructs were expressed under the control of CuSO_4_ inducible pMT promoter (Invitrogen).

### Anti-Ser antibody uptake assay

For the anti-Ser-ECD uptake assay, pMT-Ser, pMT-SerK2R and pMT-Ser^K1362R^ were transfected into S2R+-cells and expressed for 20 h at 25 °C. The cells were then transferred to coated coverslips and incubated with anti-Ser-ECD antibody for 20 min. on ice. Next the cells were washed with the ice-cold Schneider´s medium and chased for 30–60 min. at 25 °C. After the incubation at 25 °C, the cells were quickly washed with ice-cold PBS and fixed in PFA for 30 min at RT. Fixation was followed by an antibody staining under permeabilized conditions.

### Antibody staining and imaging

Antibody staining on wing imaginal discs and S2R + cells was performed according to standard protocols [46]. Antibodies used in this work are listed in the table. Alexa-Fluorochrome-conjugated secondary antibodies were purchased from Invitrogen/Molecular Probes. Images were acquired with a Zeiss AxioImager Z1 Microscope equipped with a Zeiss Apotome.2.

**Table:** List of antibodies and DNA dyes used with information on their source and concentration

Antibody (from species)	Source or Reference	Dilution
Anti Dl (mouse)	DSHB C594.9B	1:100 (surface staining)
Anti HA (rat)	Roche Clone 3F10	1:500 1:2500 (co-IP, WB)
Anti HA (rabbit)	Cell Signaling C29F4	1:1500
Anti Ser (rat)	Gift from K. Irvine	1:1000
Anti Ser (rabbit)	Gift from E. Knust	1:100
Anti N(extra) (mouse)	DSHB C458.2 H	1:100
Anti Wg (mouse)	DSHB 4D4	1:500
Anti ß-Gal (rabbit)	Cappel Research Products	1:1500
Anti Rab7 (mouse)	DSHB CG5915	1:100
Anti V5 (rabbit)	Sigma-Aldrich	1:500
Anti V5 (mouse)	Invitrogen, R961-25	1:1000 1:3000 (WB)
Anti Tubulin (mouse)	Sigma-Aldrich	1:100 000 (WB)
Anti Rabbit Alexa 488 (goat)	Invitrogen/Molecular Probes	1:500
Anti Rabbit Alexa 568(goat)	Invitrogen/Molecular Probes	1:500
Anti Rabbit Alexa 647 (goat)	Invitrogen/Molecular Probes	1:500
Anti Mouse Alexa 488 (goat)	Invitrogen/Molecular Probes	1:500
Anti Mouse Alexa 568 (goat)	Invitrogen/Molecular Probes	1:500
Anti Mouse Alexa 647 (goat)	Invitrogen/Molecular Probes	1:500
Anti Rat Alexa 488 (goat)	Invitrogen/Molecular Probes	1:500
Anti Rat Alexa 568 (goat)	Invitrogen/Molecular Probes	1:500
Anti Rat Alexa 647 (goat)	Invitrogen/Molecular Probes	1:500
Anti Rabbit HRP (goat)	Dianova	1:5000 (WB)
Anti Mouse HRP(goat)	Dianova	1:5000 (WB)
Hoechst 33,258	Sigma Aldrich	1:10.000

Western blot analysis with proteins extracted from L3 larvae was performed according to the protocol described in Berndt et al. 2017.

### Co-immunoprecipitation assay

For Co-IP, the S2R + cells were seeded in 6 x well plates and, 24 h after seeding, transfected with 1.5–2.5 µg DNA. The constructs were expressed for 20 h and subsequently the isolation of proteins was performed. Protein lysates were extracted in lysis buffer as described in [47]. Lysis buffer: 50 mM Tris-HCl, pH 7,4; 150 mM NaCl; 1% NP-40; 0,25% Sodium Deoxycholate. HA-tagged Ser proteins were precipitated with monoclonal anti-HA-coupled agarose (Pierce Anti-HA Agarose, ThermoScientific #26181).

Protein samples were separated on SDS gels and subsequently transferred to PVDF membrane. Primary and secondary antibodies used are listed in the Table.

### Ubiquitylation assay

S2 cells were transfected with Ser-V5-6×His, including WT, K2R and K1362R mutants, HA-Mib1, and Flag-Ubi in 6-well plate using Effectene (QIAGEN). The cells were treated with 100µM E-64 (Merck), 10mM NH_4_Cl, 200µM Chloroquine (Thermo Scientific Chemicals) and further incubate for 5 h at 25 °C. Each sample was then washed by PBS and lysed and homogenised in a urea lysis buffer (50mM NaHPO_4_ (pH8), 300 mM NaCl, 8 M Urea, 0.2% Tergitol 15-S-9, 10mM N-ethylmaleimide, 50µM MG-132, Halt Protease inhibitor cocktail (Thermo Fisher)) using a 25-Gauge needle. After centrifugation (for 5 min at 21 kg) to remove cell debris, 10% of each lysate was separated as an input sample, and the rest was further incubated with HisPur Ni-NTA Magnetic Beads (Thermo Fisher) for 30 min at room temperature to pull-down Ser-V5-6×His proteins. The samples were all mixed with SDS sample buffer (Thermo Fisher) supplemented with 50mM DTT and 500mM imidazole, and incubated for 10 min at 70 °C. The protein samples were separated by 3–8% NuPAGE Tris-Acetate gels and transferred to immobilon PVDF membrane (Merck), following manufacturer’s instruction. The blots were probed with following primary and secondary antibodies, and were scanned on Odyssey CLx (Licor) imaging system: Mouse anti-Flag (1/5000, M2, Merck), mouse anti-HA (1/5000, HA-7, Merck), rabbit anti-V5 (1/5000, A190-120 A, Bethyl Laboratories), rabbit anti-βActin (1/10000, 4H1, Proteintech), anti-mouse IgG Alexa Fluor Plus 800 (1/10000, A32730, Thermo Fisher), and anti-Rabbit IgG Alexa Fluor Plus 680 (1/10000, A32734, Thermo Fisher).

### Quantification of ectopic Wg activation and cis-inhibition (CI)

Relative ectopic Wg activation was calculated by measuring the lengths of anterior and/or posterior stripe of ectopic Wg expression. The sum of these lengths was divided by the length of the endogenous Wg expression along the D/V boundary to for normalization. CI was quantified as a percentage of the length of the gap in the endogenous expression of Wg relative to its length. Statistical significance was assessed using an unpaired two-sample t-test with unequal variance. Significance levels were defined as follows: *p* > 0.05, not significant (ns); *p*< 0.05, significant (*); *p < 0.01*, highly significant (**); p< 0.001, very highly significant (***). 5 discs were used for the quantification of each genotype.

#### Quantification of Western-blots

Band intensities were measured using ImageJ Fiji. Statistical analysis was performed using an unpaired two-sided students t-test.

## Supplementary Information

Additional file 1: Fig. S1. The adult phenotype of Dl^attP^-Dl-Ser-HA/Df flies. Fig. S1. Alignment of the intracellular domains of the Ser proteins from different insect species. Figure 2S2. Ubiquitylation of Ser, SerK2R and SerK1362R by Mib1 in S2R + cells. Figure 5S1. The activity of the generated Ser variants not shown in the main text. Table S1. Overview of analysed Ser-variants with information about their activity and endocytosis.

## Data Availability

No datasets were generated or analysed during the current study.

## Abbreviations

Dl: Delta
Mib1: Mindbomb1
NB: N-Box
CB: C-Box
MZM: Mib-Herc2-ZZ zinkfinger-Mib1-Herc2 domain
REP: Mib repeat-Mib repeat domain
Neur: Neuralized
Ks: Lysines
JAG1: JAGGED1
ubi: Ub iquitylation
kuz: Kuzbanian
NEXT: Notch EXtracellular Truncation
NICD: Notch intracellular domain
Su(H): Suppressor of Hairless
ICDs: Intracellular domains
wg: Wingless
R: Arginine
Dll1: Delta-like1
ptcGal4: Patched-Gal4
A/P-boundary: Anterior-posterior compartment boundary
D/V-boundary: Dorso-ventral compartment boundary
