# Supplementary material for: The intracellular domains of the DSL ligands Serrate and Delta provide different activities
Source: Cell Commun Signal. 2025 Oct 31;23:470. doi: 10.1186/s12964-025-02472-w (PMC12577249; doi:10.1186/s12964-025-02472-w)
Supplement: Supplementary file 1 — Additional file 1: Fig. S1. The adult phenotype of DlattP-Dl-Ser-HA/Df flies. Fig. S1. Alignment of the intracellular domains of the Ser proteins from different insect species. Figure 2S2. Ubiquitylation of Ser, SerK2R and SerK1362R by Mib1 in S2R + cells. Figure 5S1. The activity of the generated Ser variants not shown in the main text. Table S1. Overview of analysed Ser-variants with information about their activity and endocytosis. [file 12964_2025_2472_MOESM1_ESM.docx]

***
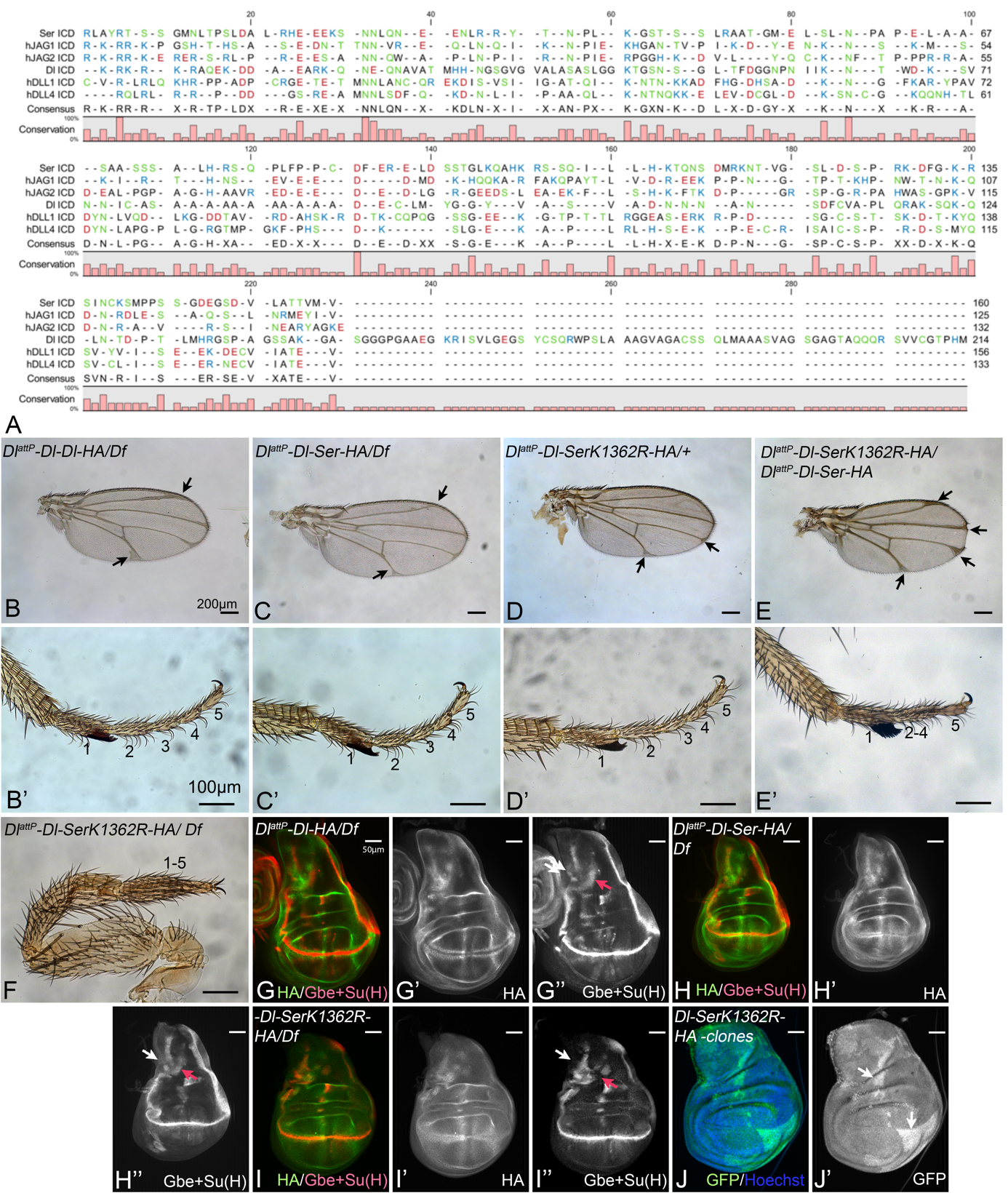
***

***Fig. S1.*** *(A) Sequence comparison of the ICDs of Ser, Dl and their human orthologs.* ***(B-F)*** *The adult phenotype of* *Dl^attP^-Dl-Ser-HA/Df flies. (B, C) The haplo-insufficient wing phenotype of Dl^attP^-Dl-HA/ Df and DlattP-Dl-Ser-HA/Df flies. Note, that the broadening of the wing vein tips (arrows) is less severe in the case of Dl^attP^-Dl-Ser-HA/Df flies. (B’, C’) Both genotypes displayed the wildtype leg phenotype. (D, D’) Adult phenotype of Dl^attP^-Dl-SerK1362-HA/+ flies. The flies displayed broadening of the vein tips very similar to Dl^attP^-Dl-HA/ Df (arrows). (E-F) The phenotype of Dl^attP^-Dl-SerK1362-HA/ Dl^attP^-Dl-Ser-HA flies displays a strong wing and leg phenotype. In the leg, the tarsal segments are fused and the whole tarsal area reduced. (G, G’, H, H’) The expression pattern of Dl^attP^-Dl-HA/Df and Dl^attP^-Dl-Ser-HA/Df in the wing imaginal disc is very similar, indicating that DlattP-Dl-Ser-HA/Df is correctly expressed. (G’’, H’’) The Notch reporter Gbe+Su(H) is similar expressed in both genotypes indicating that* Dl-Ser-HA correctly activates the Notch pathway in the disc. (I-I’’) Expression of Dl-SerK1362R in the disc is similar to Dl (compare with G). However, the expression pattern of the Notch activity reporter Gbe+Su(H) is reduced indicating a defect in Notch signalling. (J, J’) Clonal analysis of *Dl^attP^-Dl-SerK1362*. Only the wildtype orphan clone is detectable (arrows). This indicates that homozygousity of *Dl^attP^-Dl-SerK1362* is cell lethal.

**
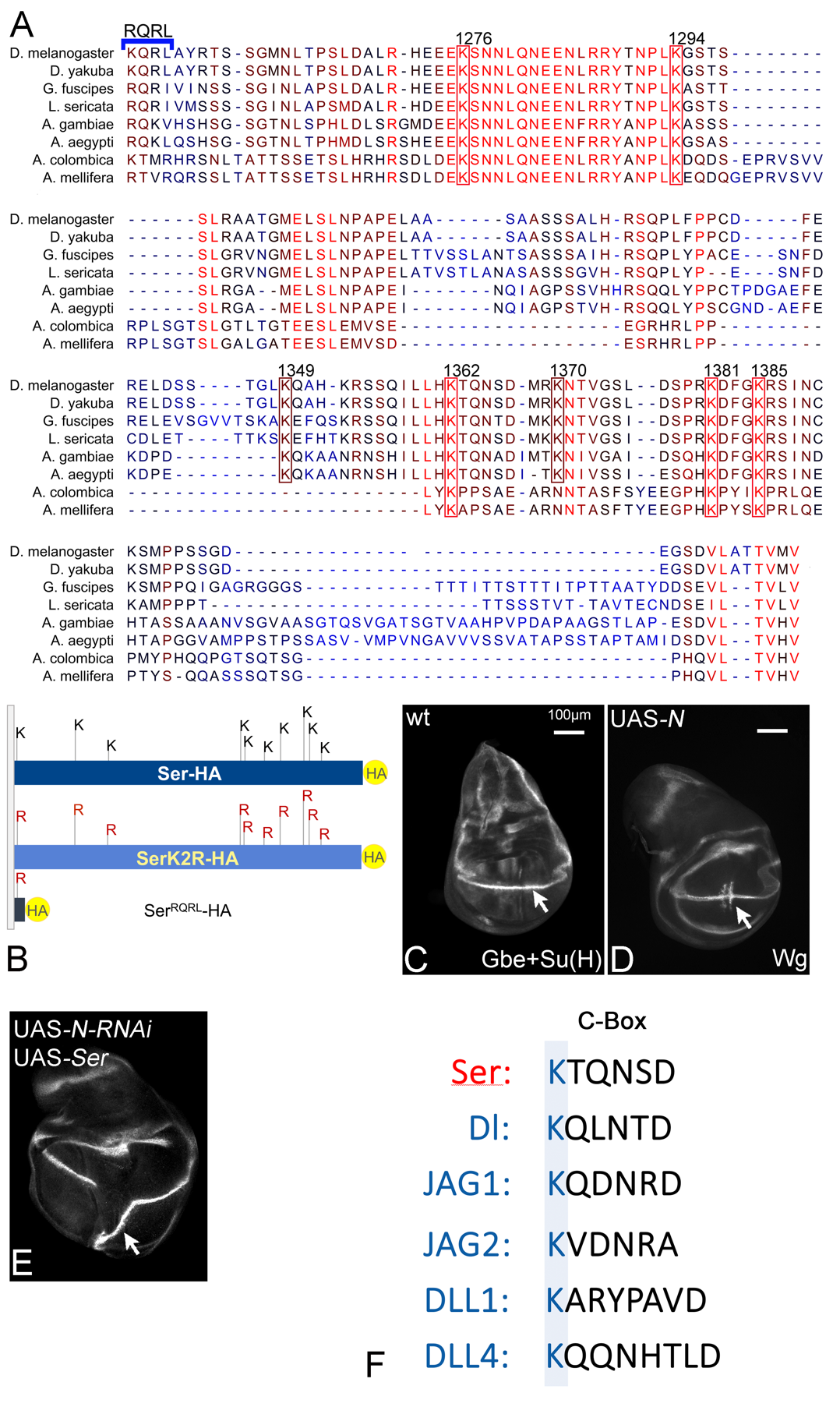
**

***Fig. S2*** *(A) Sequence alignment of the ICDs of Ser proteins of different insect species. The five most conserved Ks (core Ks) are outlined by red boxes. Blue bracket indicates the point of the RQRL truncation. The first K of Ser is changed to R to avoid ubi of the truncated Ser^RQRL^ variant. (B) Gbe+Su(H) expression in the wildtype wing imaginal disc. The arrow points to the strong expression along the D/V-boundary which occurs in the Wg expressing cells. (C) Ectopic expression of Notch with ptcGal4 induces a slight ectopic activation of Wg close to the D/V boundary (arrow). (D) Co-expression of Ser and N-RNAi. The anterior stripe of ectopic expression is lost. The arrow points to the posterior stripe, which is unaffected by the depletion of Notch as it is induced outside the ptc domain in the adjacent posterior boundary cells. (E) Comparison of the predicted CB sequences of DSL ligands. Note, that the functionality is confirmed only for JAG1 and Dl.*


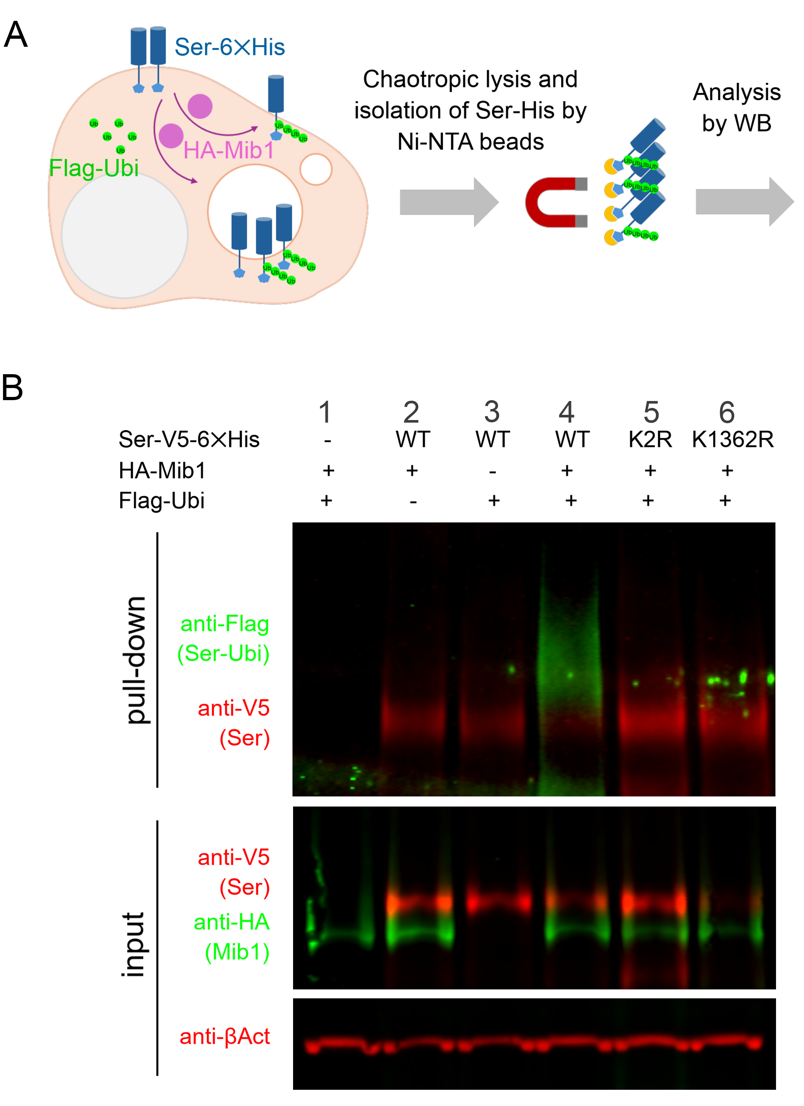


***Fig. S3****. Ubiquitylation of Ser, SerK2R and SerK1362R by Mib1 in S2R+ cells. (A) Design of the assay. Ser-V5-6×His, including WT, K2R and K1362R mutants, HA-Mib1, and Flag-Ubi were co-expressed in S2 cells. The cells were lysed with a urea lysis buffer, and Ser protein was isolated using Ni-NTA-resin. Western blotting was performed to analyse the ubiquitylation of Ser. (B) Ubiquitylation assay of Ser. The upper panel shows the pull-down samples of Ser (WT, K2R, and K1362R), where anti-Flag and V5 antibodies were used to detect Ubiquitin (green) and Ser (red), respectively. The corresponding 10% input samples are shown in the lower panel, in which Ser (red) and Mib1 (green) expression were detected by anti-V5 and HA antibodies. Representative result (n=3). Only Ser is efficiently ubiquitylated (green smear in lane 4), while SerK2R and also SerK1362R not (lanes 5 and 6). Ubiquitylation of Ser by Mib1*

*
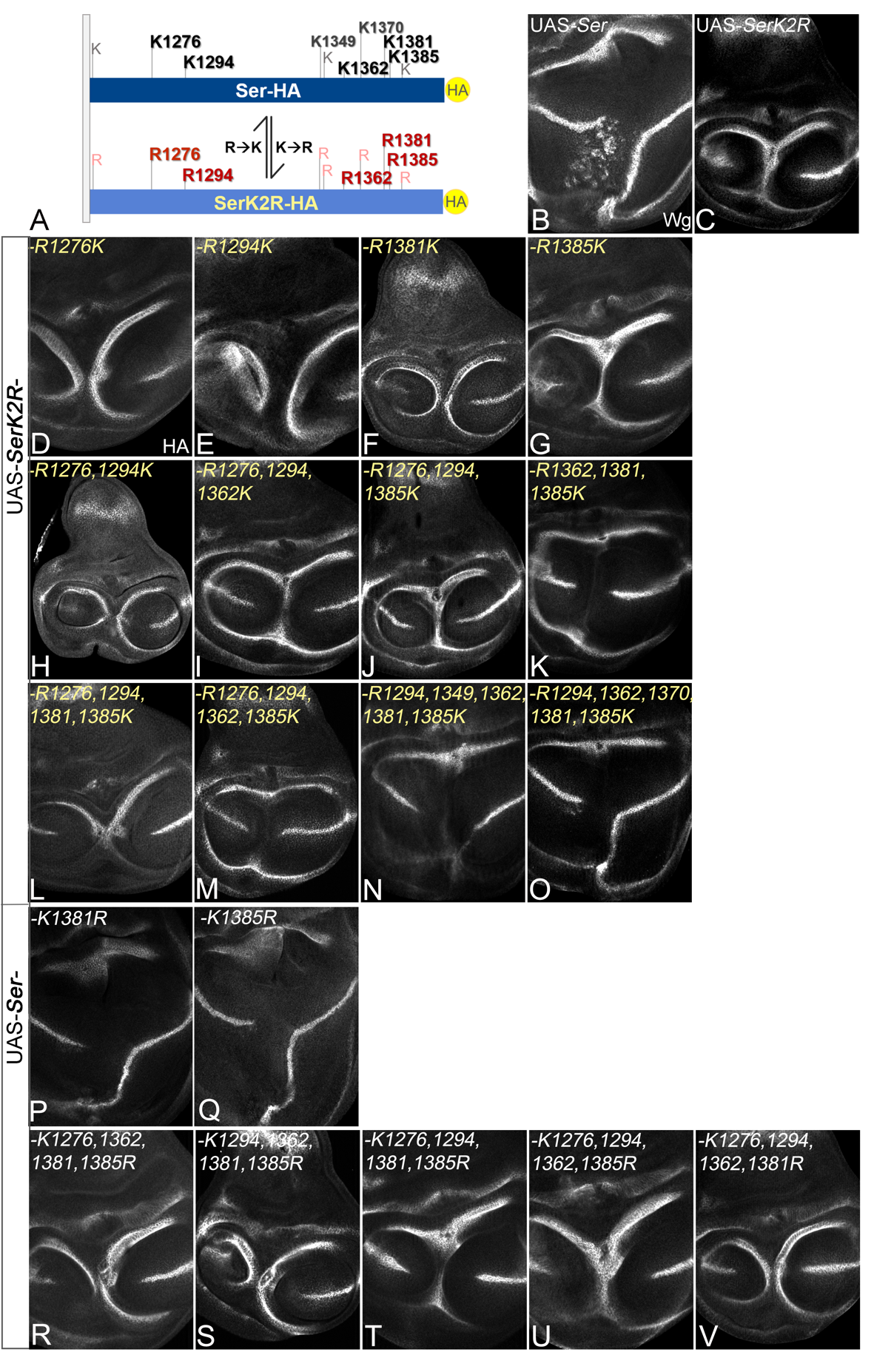
*

***Fig. S4.*** *The activity of the generated Ser-variants not shown in the main text. Expression occurred with ptcGal4. For more information, see text and for a summary of the activity, see also table 1.*

***Table S1:*** *Overview of analysed Ser variants with information about their activity and endocytosis.*

| UAS-Ser variant | Trans-  Activation | Cis-  Inhibition | Dominant  negative | Endocytosis |
| --- | --- | --- | --- | --- |
| Ser-HA | ++++ | ++ | ― | ++ |
| Ser^K1276R^-HA | ++++ | +++ | ― | + |
| Ser^K1294R^-HA | ++++ | + | ― | n.a. |
| Ser^K1362R^-HA | ― /+ | ++++ | ― | ―/+(?) |
| Ser^K1370R^-HA | ++++ | + | ― | n.a. |
| Ser^K1381R^-HA | ++++ | +++ | ― | + |
| Ser^K1385R^-HA | ++++ | +++ | ― | + |
| Ser^K1276,1294R^-HA | ++ | +++ | ― | + |
| Ser^K1362,1381,1385R^-HA | ― | ++++ | ― /+ | n.a. |
| Ser^K1276,1294,1362,1381R^-HA | ― |  | ++++ | ― |
| Ser^K1276,1294,1362,1385R^-HA | ― |  | ++++ | ― |
| Ser^K1276,1294,1381,1385R^-HA | ― |  | ++++ | ―/+(?) |
| Ser^K1276,1362,1381,1385R^-HA | ― |  | ++++ | ― |
| Ser^K1294,1362,1381,1385R^-HA | ― |  | ++++ | n.a. |
| Ser^K1276,1294,1362,1381,1385R^-HA (Ser^5R^) | ― |  | ++++ | ― |
| SerK2R-HA | ― |  | ++++ | ― |
| SerK2R^R1276K^-HA | ― |  | ++++ | ― |
| SerK2R^R1294K^-HA | ― |  | ++++ | ― |
| SerK2R^R1362K^-HA | ― |  | ++++ | n.a. |
| SerK2R^R1381K^-HA | ― |  | ++++ | ― |
| SerK2R^R1385K^-HA | ― |  | ++++ | ― |
| SerK2R^R1276,1294K^-HA | ― |  | ++++ | n.a. |
| SerK2R^R1276,1294,1362K^-HA | ― |  | ++++ | ― |
| SerK2R^R1276,1294,1385K^-HA | ― |  | ++++ | n.a. |
| SerK2R^R1362,1381,1385K^-HA | ― |  | + | n.a. |
| SerK2R^R1276,1294,1362,1381K^-HA | ― |  | ++ | ―/+(?) |
| SerK2R^R1276,1294,1362,1385K^-HA | ― | ++++ | ― | ― |
| SerK2R^R1276,1294,1381,1385K^-HA | ― |  | ++ | n.a. |
| SerK2R^R1276,1362,1381,1385K^-HA | ― | ++++ | ― | n.a. |
| SerK2R^R1294,1362,1381,1385K^-HA | ―/+ | ++++ | ― | ―/+(?) |
| SerK2R^R1276,1294,1362,1381,1385K^-HA (SerK2R^5K^) | ++++ | + | ― | + |
| SerK2R^R1294,1362,^**^1370^**^,1381,1385K^-HA | ++++ | +++ | ― | + |
| SerK2R^R1276,1294,1362,^**^1370^**^,1381K^ -HA | ++++ | +++ | ― | + |
| SerK2R^R1294,^**^1349^**^,1362,1381,1385K^-HA | ― /+ | ++++ | ― | n.a. |
| SerK2R^R1276,1294,^**^1349^**^,1362,1381K^-HA | + | ++++ | ― | ―/+(?) |
| SerK2R^R1276,1294,1362,1370,1381,1385K^ -HA | ++++ | + | ― | + |
| Ser^RQRL^-HA | ― |  | ++++ | + |
